# Supplementary material for: Molecular heterogeneity and early metastatic clone selection in testicular germ cell cancer development
Source: Br J Cancer. 2019 Feb 11;120(4):444–52. doi: 10.1038/s41416-019-0381-1 (PMC6461884; doi:10.1038/s41416-019-0381-1)
Supplement: Supplementary file 9 — Supplementary Methods [file 41416_2019_381_MOESM9_ESM.pdf]

Supplementary Methods

Patient characteristics:

	Age at diagnosis	diagnosis	TNM stage	stage	Initial treatment	metastases	remark
T3209	15 y	EC, TE, YST	T3N1M1b	IIIc	4xBEP + 2xVIP	multiple metastases and (late) relapses	
T6107	21 y	TE, YST, EC	T2N3M1a	IIIa	3xBEP + 2xEP + 3xBOP	multiple metastases and relapses	karyotype*
T1382	36 y	EC, TE, YST	T3N1M1b	IIIc	4xBEP	multiple metastases and relapses	DoD
T618	20 y	TE, YST	T3N1M1b	IIIc	4xBEP	multiple metastases and relapses	DoD

Abbreviations: (B)EP = (Bleomycin) / Etoposide / Cisplatin; VIP = Etoposide / Ifosfamide / Cisplatin, BOP = Bleomycin / Vincristine / Cisplatin; DoD = death of disease. Karyotype primary tumor (64, X, -Y, +X,+1, +2, +3, +6, +7, +7,+8, +8, +12, +i(12(p10)x2, +i(14)(q10), +15, +der(16), +t(6;16)(q14 or q15; q24), +17, +19, +21, +21, 2-10 mar [Cp6], 6 cells) obtained after short term *in vitro* culture using standard procedures (1).

Preparation of patient samples

DNA and RNA from frozen tumor tissue were prepared using standard procedures as described previously (2). Peripheral blood or adjacent normal testis tissue was used as normal control. FFPE metastases samples were macro-dissected to remove normal adjacent tissue and processed for DNA preparation as described before (3). Purified tumor components, as defined by an experienced pathologist (JWO), were isolated from frozen tissue slices after staining for alkaline phosphatase enzyme reactivity (dAP) on GCNIS and EC cells (4), using PALM micro-dissection (Zeiss). Tissues were processed using Proteinase K digestion and purified with Cell lysis solution (Promega) with 5% Chelex 100 Resin (BioRad) (5) or the QIAamp DNA micro Kit (following the protocol for laser-microdissected tissues, Qiagen), and quantified with Qubit2.0 (ThermoFisher Scientific). The YST metastatic sample was processed at DKFZ in Heidelberg (FRG) using the Promega Maxwell automated extraction system for FFPE samples. Sample information is provided in Supplementary Table S1.

Whole genome and targeted DNA sequencing.

Tumor and paired normal DNA samples were whole genome sequenced at 40times coverage and analyzed at Complete Genomics Inc. (CG) (Mountain View, CA, USA) using NCBI build 36.3 as human reference genome and pipeline software version 2.0.2.22 (6). All data are available under ENA PRJEB20644, accession numbers ERX2100523 - 530:

Targeted NGS was performed by semiconductor sequencing with the Ion Torrent Personal Genome Machine (PGM) with supplier's materials and protocols (ThermoFisher Scientific) as previously described (5). In brief, genomic DNA input varied between 0.2 and 10 ng, depending on the amount of tissue available. Amplicon primer sets (Supplementary Table S3) for selected somatic SNV (n=127), putative breakpoints (n=30) and informative regions across the genome (n=173), were designed with Ion AmpliSeq™ Designer. Library preparations were performed with the AmpliSeq Library Kit 2.0-384 LV. Number of PCR cycles varied depending on the amount of input and DNA source (frozen: 18 cycles; FFPE: 21 cycles above 1ng input or 24 cycles). Template preparation and sequencing were done with the Ion PGM Hi-Q Chef Kit on an Ion 318v2 chip. Sequence information of experiments with a median coverage of 250 to 3000 reads was analyzed with Variant Caller v4.4.2.1 (ThermoFisher Scientific) and variants were annotated in a local Galaxy pipeline using ANNOVAR (human genome build 37, COSMIC68, CLINVAR, dbsnp138NonFlagged, ESP6500si_ALL, 1000g2012apr_ALL, 1000g2012apr_EUR) (http://www.openbioinformatics.org/annovar/). Sequence data are available under ENA PRJEB20644, accession numbers: ERX2019898-958.

DEPArray experiments were performed by Menarini Silicon Biosystems (Castel Maggiore, Italy), essentially as described previously (7). Cells were dissociated from FFPE sections of cases T6107 and T618 following antigen retrieval, stained with Keratin-Alexa488, Vimentin-Alexa647 and DAPI, and purified on the DEPArray™ system. Genomic DNA from purified cell populations was subjected to LowPass WGS and copy number profiles were generated with Control-FREEC software (8).

RNA analyses.

RNA samples were rRNA reduced with RiboZero Gold and Ion Proton sequenced using the Ion Total RNA-seq kit, the Ion PI Template OT2 200 Kit v3 and the Ion PI Sequencing 200 Kit v3 on a PI chip. Average read length was 90 bases and approximately 50 million mapped reads were obtained. Primary data analysis was performed using the Torrent_Suite software vs 4.0.2 and Homo sapiens hg19. Data are available from ArrayExpress, accession number: E-MTAB-5746.

Methylation analysis.

Generation of methylation profiles of primary tumor genomic DNA was performed as previously described (9) or at the Microarray unit of the Genomics. T6107 YST metastasis genomic DNA was processed at the Proteomics Core Facility of the German Cancer Research Center (DKFZ, Heidelberg) strictly adhering to the Illumina protocols (EPIC). Copy number alterations were resolved using the Conumee package (10). Data are available from GEO (GSE58538, GSM1413103 to GSM1413106) or from ArrayExpress (E-MTAB-5842, sample T6107-YSTmeta).

Mutation-specific Q-PCR.

Primers sets designed for specific detection of putative somatic SNV were used for quantitative PCR. 10 ng of genomic DNA was amplified using wild type and mutation specific primers (Supplementary Table S2) in 20 µl reactions containing Absolute SybrRox mix (AB1163) in a 7500Fast thermocycler for 35 cycles at different annealing temperatures (optimal 63-66°C). The presence of a somatic SNV was called when at least 5 Ct difference was

observed with control samples and the appropriate DNA fragment length was observed on agarose gel.

Bioinformatic analyses.

The Complete Genomics output masterVarBeta files were filtered for Only A or Mismatch, SQHIGH, Alle1 Normal <=2&<10% (#T618, <=3&<10%), Tumor reads>20, Normal reads>10, and RepeatMasker negative. In the absence of additional bioinformatics tools, a total of 2142 candidate somatic SNV positions were verified by visual inspection of the aligned reads in IGV (11) (http://software.broadinstitute.org/software/igv/home). Criteria for exclusion of variants were: sequence quality insufficient, very few variant reads with identical start position, variant also observed in any of the other three tumor/normal pairs. Variants retained (n=1239) are listed in Supplementary Table S4. These variants were annotated by the CG pipeline (human genome build 36.3, dbsnp130, COSMIC v48) and functional consequences were predicted using Annovar and Alamut software (version 2.2 http://www.interactive-biosoftware.com/) (Supplementary Table S6). Potential structural variants called by CG (SomaticHighConfidenceJunctionsBeta T-N) were filtered for events occurring in a normal population (base line set) and further investigated for generation of gene fusions with iFuse (http://ifuse.erasmusmc.nl/; (12)). The CG-assembled junction sequences (Supplementary Table S5) were re-aligned against the hg19 human reference sequence (BLAT, http://genome-euro.ucsc.edu/). Normalized relative read coverage and best lesser allele frequencies (LAF, per 100 kb segment, somaticCnvDetailsNondiploidBeta file) were plotted using the tools in Galaxy (http://bioinf-galaxian.erasmusmc.nl/galaxy/).

SNP calls from targeted sequencing experiments were inspected in IGV. Only those positions showing proper alignment, sufficient coverage and heterozygosity (40-60%) in the matched normal sample were included in the analyses of dissected tumor specimens. For a

given sample, the SNP allele frequency was used when total coverage was at least 20 reads, and the minimal number of somatic SNV reads was required to be at least 4. In order to distinguish clonal variation, lesser allele frequencies were determined for the heterozygote positions in the sequenced regions (Supplementary Table S7). Lesser allele frequencies were plotted using R scripts to visualize the alterations. The presence of specific breakpoints was assessed by constructing an artificial reference sequence for the predicted sequence fusions using the CG-assembled fusion sequence (Supplementary Table S5). For these fusions, only their presence in the specimen was scored when at least 3 matching reads were observed. For the comparison of different specimens of a particular case, the LAF frequencies averaged per amplicon (Supplementary Table S8) were plotted using the enhanced heat map plotting function (heatmap.2) in R. The numbers of RNA-seq reads carrying a somatic mutation at a specific position were calculated using an R script for counting Bam files reads.

For mutational signature calling, the trinucleotide context of each somatic SNV was derived using the appropriate human reference genome (build hg18 or hg19) and enumerated into a mutational spectrum matrix Mij (i = 96; number of trinucleotide contexts; j = number of samples) using the SomaticSignatures R package (v2.8.4) (13). Established mutational signatures were downloaded from COSMIC (http://cancer.sanger.ac.uk/cancergenome/assets/signatures_probabilities.txt). Per sample, a constrained linear combination of the validated mutational signatures was constructed, which best reconstructs the sample-specific mutational spectrum, using non-negative least squares regression implemented in the MutationalPatterns R package (v1.0) (14). The statistical analyses were conducted using the R statistical package version 3.3.0. R-scripts used for this study are available on request.

TargetClone: Assessment of the evolutionary relationships between different tumor samples from a particular case relies predominantly on calculating the number of chromosomal copy number changes and the acquirement of somatic SNV (15). Both the absence of a large

series of normal samples run on the same platform and the variable quality of the input DNA samples did not allow for estimating chromosome copy numbers using various published algorithms based on read coverage. The tool TargetClone was designed to reconstruct evolutionary trees for multiple samples of a cancer and to predict copy numbers, alleles and tumor fractions in every sample (16). Compared to other methods, TargetClone does not rely on read depth information, but instead uses allele frequencies and somatic SNV. Two main assumptions are implemented. First, the samples are assumed to be as homogeneous as possible, containing a single cancer subclone and possibly normal cell contamination. Second, horizontal and vertical dependencies are assumed to exist between samples. Thus, two adjacent measurements on a chromosome or chromosomal region are expected to be affected by the same event (horizontal dependency). Also, it is expected that samples with highly overlapping mutations are closely related (vertical dependency). These dependencies allow more accurate estimation of the evolutionary distance between samples. The evolutionary trees were reconstructed using the default settings of TargetClone and taking a tetraploid precursor as starting point for the trees. In order to get biological relevant trees (Fig. 3), the differentiated tumor components should be descendants from an EC type precursor cell.

References

1. van Echten J, Oosterhuis JW, Looijenga LH, van de Pol M, Wiersema J, te Meerman GJ, et al. No recurrent structural abnormalities apart from i(12p) in primary germ cell tumors of the adult testis. Genes Chromosomes Cancer. 1995;14(2):133-44. Epub 1995/10/01.
2. van der Zwan YG, Rijlaarsdam MA, Rossello FJ, Notini AJ, de Boer S, Watkins DN, et al. Seminoma and embryonal carcinoma footprints identified by analysis of integrated genome-wide epigenetic and expression profiles of germ cell cancer cell lines. PLoS ONE. 2014;9(6):e98330. Epub 2014/06/03.
3. Looijenga LH, Gillis AJ, Verkerk AJ, van Putten WL, Oosterhuis JW. Heterogeneous X inactivation in trophoblastic cells of human full-term female placentas. American journal of human genetics. 1999;64(5):1445-52. Epub 1999/04/17.
4. Stoop H, Kirkels W, Dohle GR, Gillis AJ, den Bakker MA, Biermann K, et al. Diagnosis of testicular carcinoma in situ '(intratubular and microinvasive)' seminoma and embryonal carcinoma using direct enzymatic alkaline phosphatase reactivity on frozen histological sections. Histopathology. 2011;58(3):440-6. Epub 2011/02/18.

5. Dubbink HJ, Atmodimedjo PN, Kros JM, French PJ, Sanson M, Idbaih A, et al. Molecular classification of anaplastic oligodendroglioma using next-generation sequencing: a report of the prospective randomized EORTC Brain Tumor Group 26951 phase III trial. Neuro Oncol. 2016;18(3):388-400. Epub 2015/09/12.
6. Drmanac R, Sparks AB, Callow MJ, Halpern AL, Burns NL, Kermani BG, et al. Human genome sequencing using unchained base reads on self-assembling DNA nanoarrays. Science. 2010;327(5961):78-81. Epub 2009/11/07.
7. Bolognesi C, Forcato C, Buson G, Fontana F, Mangano C, Doffini A, et al. Digital Sorting of Pure Cell Populations Enables Unambiguous Genetic Analysis of Heterogeneous Formalin-Fixed Paraffin-Embedded Tumors by Next Generation Sequencing. Scientific reports. 2016;6:20944. Epub 2016/02/13.
8. Boeva V, Popova T, Bleakley K, Chiche P, Cappo J, Schleiermacher G, et al. Control-FREEC: a tool for assessing copy number and allelic content using next-generation sequencing data. Bioinformatics. 2012;28(3):423-5. Epub 2011/12/14.
9. Rijlaarsdam MA, Tax DM, Gillis AJ, Dorssers LC, Koestler DC, de Ridder J, et al. Genome wide DNA methylation profiles provide clues to the origin and pathogenesis of germ cell tumors. PLoS One. 2015;10(4):e0122146. Epub 2015/04/11.
10. Hovestadt V, Zapatka M. conumee: Enhanced copy-number variation analysis using Illumina DNA methylation arrays. R package version 1.6.0. http://bioconductororg/packages/conumee/. 2015.
11. Robinson JT, Thorvaldsdottir H, Winckler W, Guttman M, Lander ES, Getz G, et al. Integrative genomics viewer. Nat Biotechnol. 2011;29(1):24-6. Epub 2011/01/12.
12. Hiltemann S, McClellan EA, van Nijnatten J, Horsman S, Palli I, Teles Alves I, et al. iFUSE: integrated fusion gene explorer. Bioinformatics. 2013;29(13):1700-1. Epub 2013/05/11.
13. Gehring JS, Fischer B, Lawrence M, Huber W. SomaticSignatures: inferring mutational signatures from single-nucleotide variants. Bioinformatics. 2015;31(22):3673-5. Epub 2015/07/15.
14. Blokzijl F, Janssen R, Van Boxtel R, Cuppen E. MutationalPatterns: an integrative R package for studying patterns in base substitution catalogues. http://biorxivorg/content/early/2016/10/17/071761. 2016.
15. Deshwar AG, Vembu S, Yung CK, Jang GH, Stein L, Morris Q. PhyloWGS: reconstructing subclonal composition and evolution from whole-genome sequencing of tumors. Genome Biol. 2015;16:35. Epub 2015/03/19.
16. Nieboer MM, Dorssers LCJ, Straver R, Looijenga LHJ, de Ridder J. TargetClone: A multi-sample approach for reconstructing subclonal evolution of tumors. PLoS ONE. 2018;13(11):e0208002. Epub 2018/11/30.
