# Supplementary material for: Molecular heterogeneity and early metastatic clone selection in testicular germ cell cancer development
Source: Br J Cancer. 2019 Feb 11;120(4):444–52. doi: 10.1038/s41416-019-0381-1 (PMC6461884; doi:10.1038/s41416-019-0381-1)
Supplement: Supplementary file 10 — Information Supplementary Tables S1-S8 [file 41416_2019_381_MOESM10_ESM.pdf]

Supplementary Tables description

Supplementary Table S1. Sample description used for targeted sequencing. Sample histology and origin (frozen/FFPE), isolation procedure, DNA input quantity and DNA sequence bam file codes are provided.

Supplementary Table S2. Amplification primers used for Q-PCR of wild-type and mutated targets. Primer codes refer to Supplementary Table S4, column “PCR”.

Supplementary Table S3. Amplification primers designed for Ion Torrent targeted sequence analyses. Target names refer to Supplementary Table S4, column “Target Code”.

Supplementary Table S4. Listing of all SNV from the masterVarBeta files meeting the criteria (Supplementary Methods). CG output information is provided for each SNV. Specific SNV selected for validation by mutation-specific Q-PCR (column “PCR”) or targeted sequencing analyses (column “Target Code”) are indicated. Evaluation of the RNA-seq data from T3209 and T6107 is also provided. The validation result is summarized in the “Confirmation Status” column.

Details:

Mutation–specific PCR confirmed 27 SNV. The majority of the successfully sequenced amplicons (n=127) contained the expected SNV (n=120, 94.5%). Furthermore, RNAseq of total RNA available for two tumors (T3209 and T6107) provided transcripts reads across 106 positions containing putative SNV at the DNA level. The majority of these positions identified only ref-type sequences (n=69), while mutated sequences were recovered for 37 SNV in variable read frequencies.

Supplementary Table S5. Listing of all structural variants present in the SomaticHighConfidence JunctionsBeta T-N were included when not present in the normal population. Flanking genes identified by iFUSE are indicated. CG assembled sequences could be re-aligned against hg19 reference sequence using BLAT (N=92). The column “Note” describes the predicted result of the fusion. The column “Validation” indicates fusion targets included in the targeted sequencing experiments and the result of the analyses.

Supplementary Table S6. Characteristics of all SNV validated. Annovar annotation and Alamut software prediction on functional consequences are provided. The column “Summary Prediction” counts the number of tool predictions which suggest functional consequences of the mutation. A high score suggests that the mutation may impact the biology of the cell.

Supplementary Table S7. LAF data Ion Torrent sequencing. Each sheet provides the lesser allele frequencies (LAF) of the heterozygote positions and the read frequencies of the SNV for all samples studied for each case. Missing data are given as NA. The last two columns provide the CG bestLAF and relative coverage (both averaged over 100 kb intervals, see Fig. S1) at that chromosomal position.

Supplementary Table S8. LAF data averaged per amplicon. Each sheet provides the amplicon average LAF data per case ordered according chromosomal position. At the bottom of the tables, the read coverage of SNV and the presence call for structural variants is given. Multiple measurements for PBL and primary tumor have been averaged. The last two columns provide the CG bestLAF and relative coverage (both averaged over 100 kb intervals, see Fig. S1) at that chromosomal position.
