# Supplementary material for: Molecular heterogeneity and early metastatic clone selection in testicular germ cell cancer development
Source: Br J Cancer. 2019 Feb 11;120(4):444–52. doi: 10.1038/s41416-019-0381-1 (PMC6461884; doi:10.1038/s41416-019-0381-1)
Supplement: Supplementary file 11 — Supplementary Figures S1-S10 and details [file 41416_2019_381_MOESM11_ESM.pdf]

Supplementary Figures, legends and summary

Fig. S1A: T3209

Fig. S1B: T6107

Fig. S1C: T618

Fig. S1D: T1382

Fig. S1. Summary of whole genome sequencing data. Galaxy plotting (https://bioinf-galaxian.erasmusmc.nl/galaxy/) of Complete Genomics normalized read coverage (black) and best LAF (red) data averaged over 100kb intervals, and read frequencies of SNV (blue) (hg18). The positions of the amplicons selected for targeted sequencing (Supplementary Table S3) are shown above the x-axis (purple).

Details:

SNV presented were verified in IGV using the following exclusion criteria: sequence quality insufficient, very few variant reads with identical start position, or variant also observed in any of the other three tumor/normal pairs. Variants retained (n=1239, including 47 indels) are listed in Supplementary Table S4.

High read coverage, was observed for the short arm of chromosome 12 (12p) in all four cases, in line with the expectation. Other regional high copy amplicons were rare (chr1q, 7q, and 11q in T618, and chr22q in T1382, Fig. S1).

Validation of the findings of the WGS was performed using mutation-specific quantitative PCR, analysis of RNAseq data and targeted resequencing for a panel of amplicons covering 131 SNV (including most variants with putative consequences on the encoded protein). In total, the presence of 150 SNV out of 158 tested (94.9%, Supplementary Table S4) could be confirmed. Of the 15 genomic breakpoints investigated, nine fusion events were confirmed by the targeted sequencing of the tumor DNA samples (Supplementary Table S5). Apart from mutations within exons of noncoding RNA genes, only 48 SNV may have affected the encoded protein (including mutations in proximity of a splice site) in these four cases (details in Supplementary Table S6). Using Annovar annotation and Alamut prediction

software, 38 SNV may be potentially pathogenic. Only four somatic SNV caused protein truncation and another 13 were predicted to be damaging by at least three algorithms.

Tumor-specific translocation and rearrangement events identified were rare and predicted fusion transcripts by iFuse (1) failed to provide attractive candidates for oncogenic activation (Supplementary Table S5). A specific chromosomal translocation (chr16:77.3M, chr4: 42.7M) was found for case T6107 (Supplementary Table S5) involving one chromosomal region previously identified by conventional karyotyping of T6107 in the nineties (t(6,16)(q14/15,q24)) (Supplementary Methods). Of this tumor, relative chromosome copy numbers (Figs. S1B & S2) were in good agreement with the classic karyotype analysis of six metaphase cells obtained after short term *in vitro* culture (see Supplementary Methods).

Read frequencies of SNV in these cases were generally below 40% (>95% of the SNV, Supplementary Table S4).

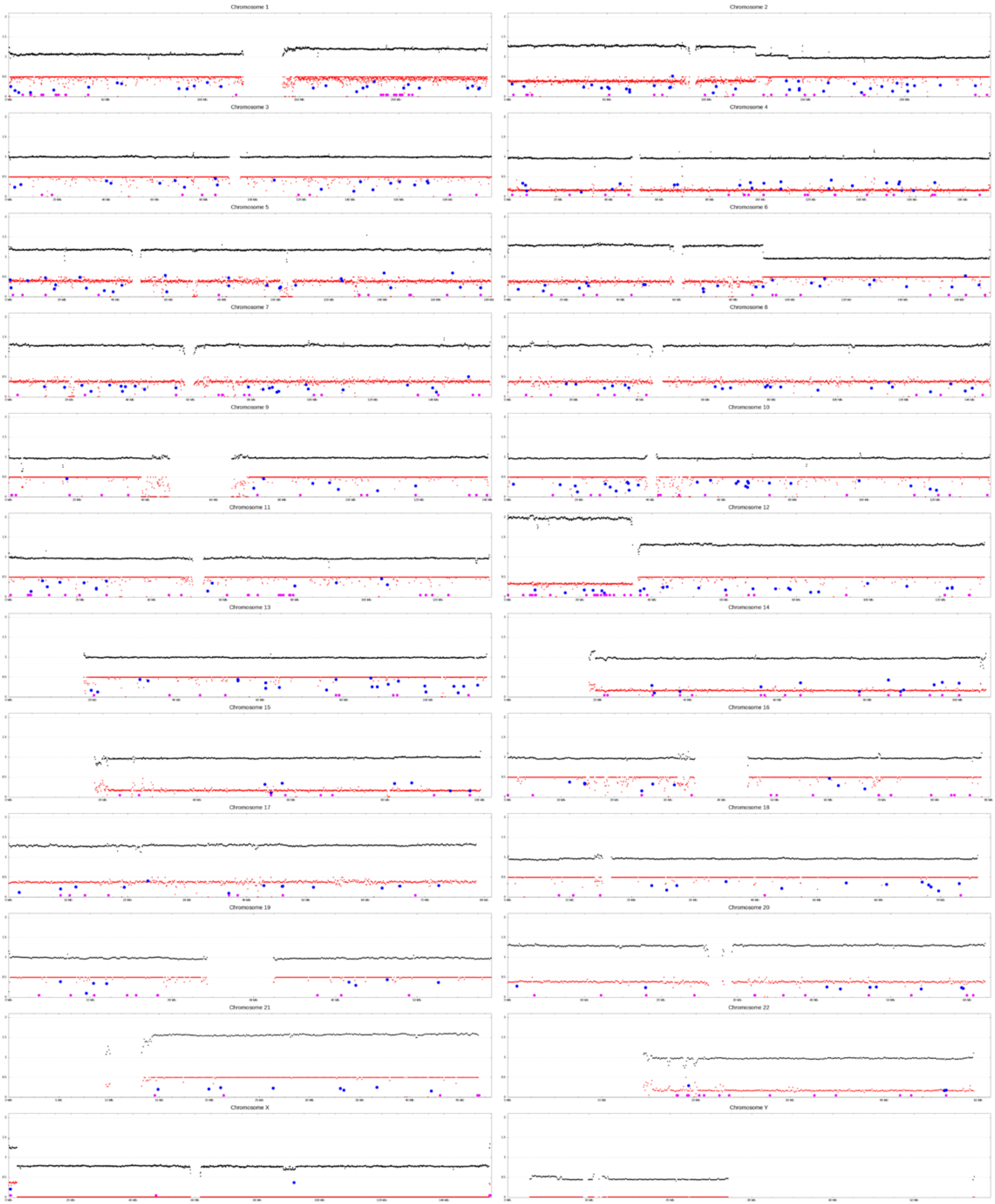

Fig. S1A, T3209

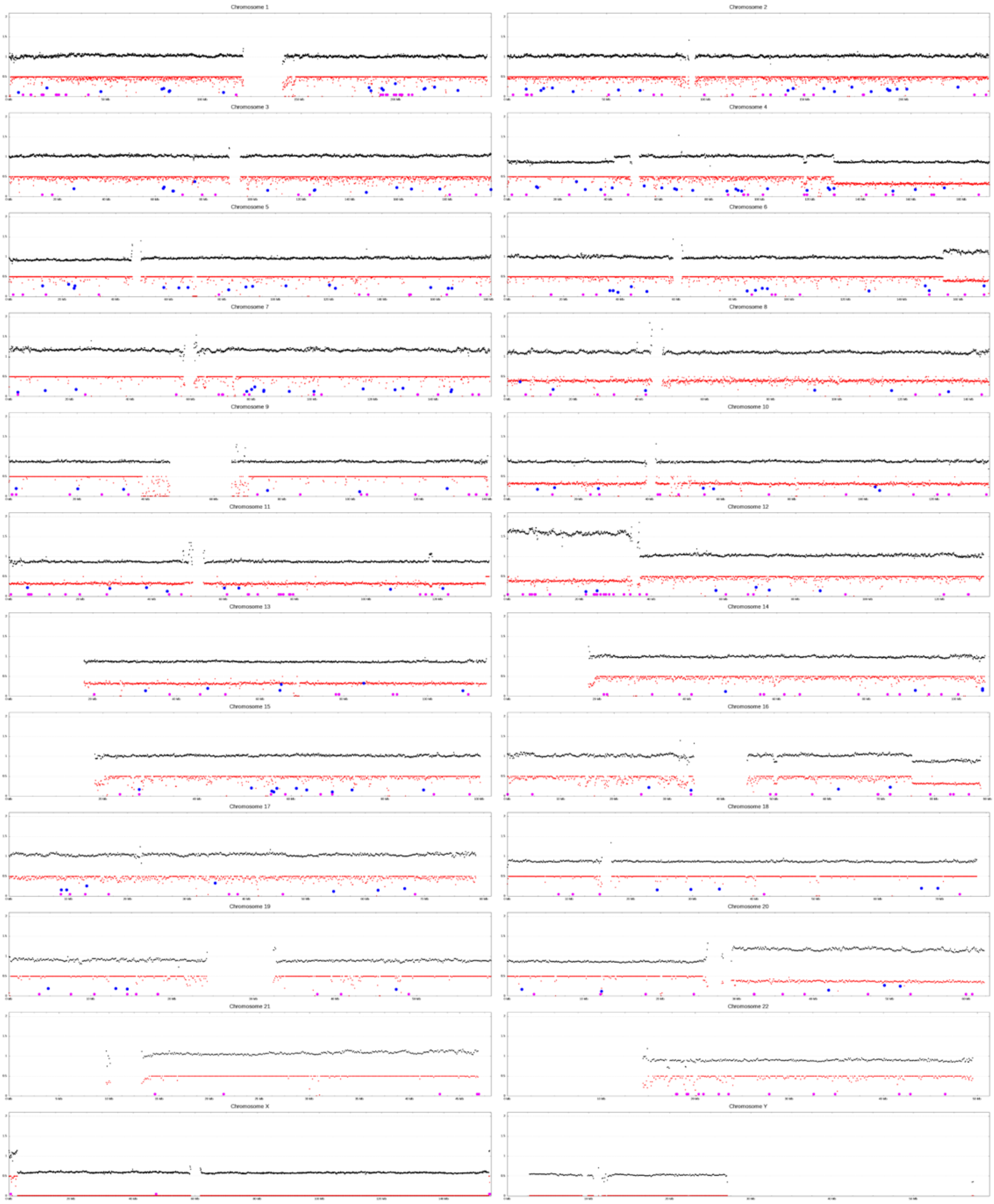

Fig. S1B, T6107

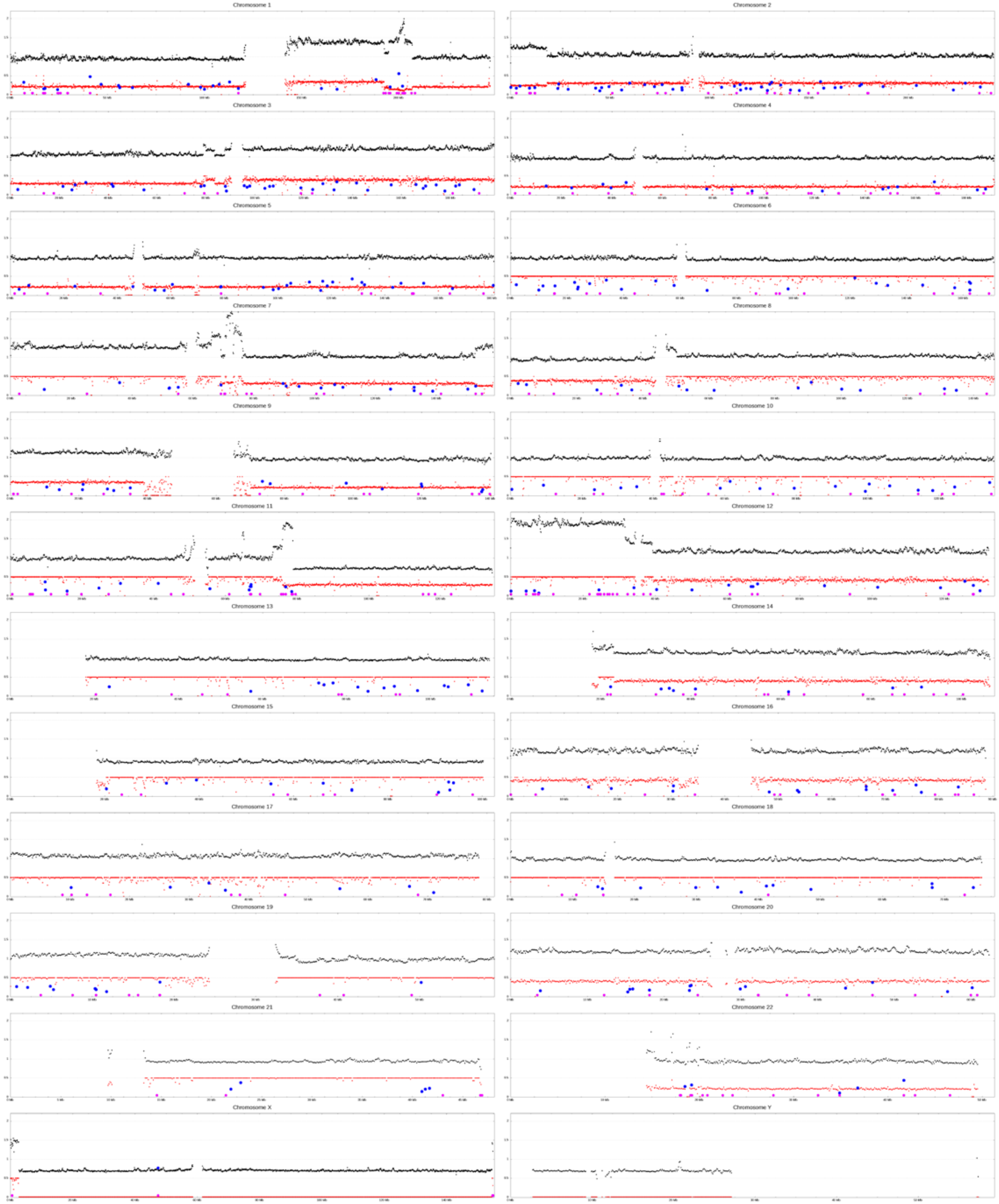
Chromosome 1
Chromosome 2
Chromosome 3
Chromosome 4
Chromosome 5
Chromosome 6
Chromosome 7
Chromosome 8
Chromosome 9
Chromosome 10
Chromosome 11
Chromosome 12
Chromosome 13
Chromosome 14
Chromosome 15
Chromosome 16
Chromosome 17
Chromosome 18
Chromosome 19
Chromosome 20
Chromosome 21
Chromosome 22
Chromosome X
Chromosome Y

Fig. S1C, T618

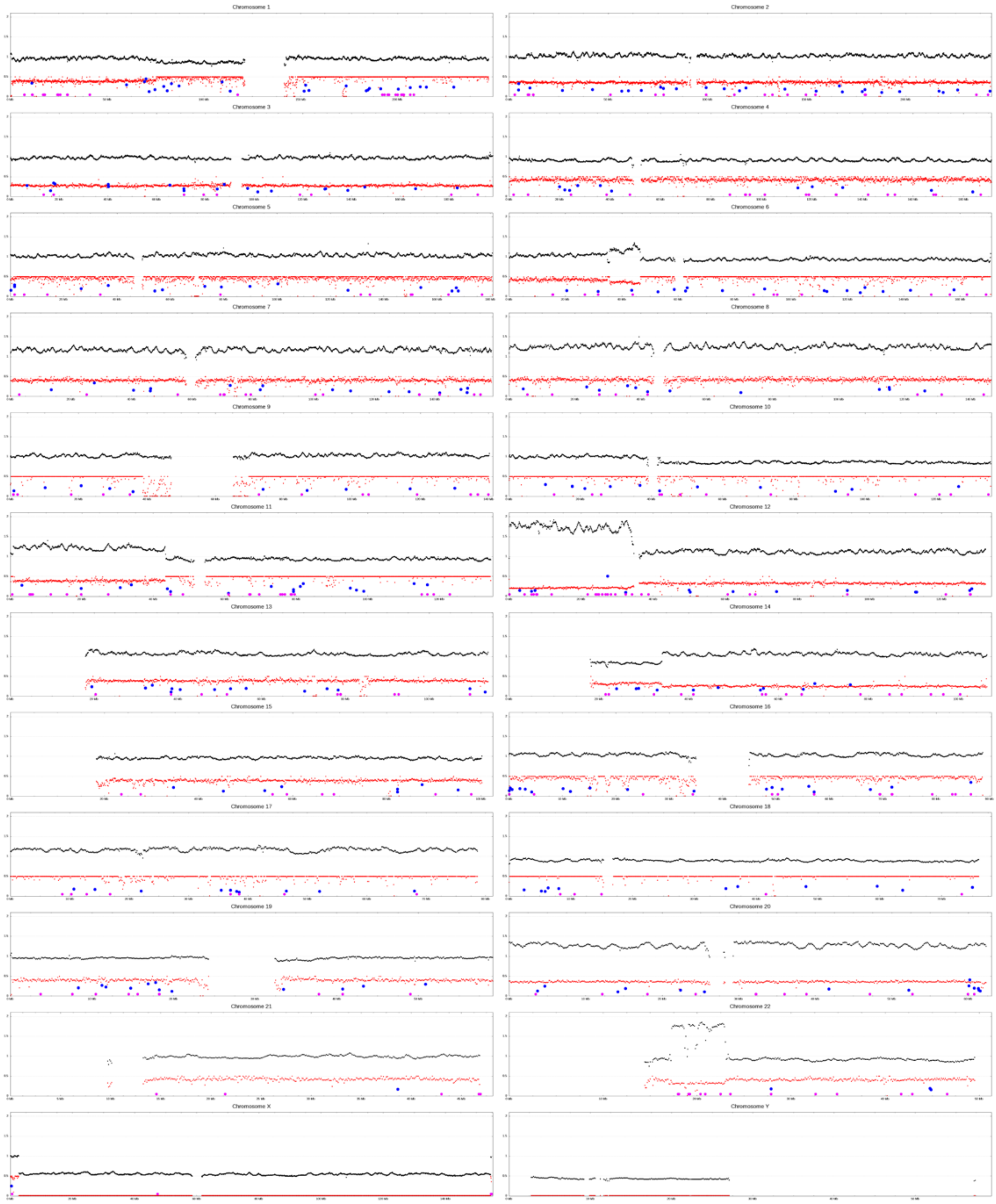

Fig. S1D, T1382

Fig. S2. Copy number variation derived from methylation array intensities. Methylation intensities from Illumina 450K methylation arrays of primary tumor genomic DNA were converted into relative copy numbers using the Conumee package(2). Red indicates reduced copy numbers, green increased copy numbers of individual chromosomal regions. Blue lines represent segments with similar copy numbers. Data are from GSM1413103 (T6107), GSM1413104 (T1382), GSM1413105 (T3209) and GSM1413106 (T618).

Details:

The methylation array intensities provided relative copy number profiles which are very well comparable to the WGS normalized read coverage profiles (Fig. S1)

Fig. S2

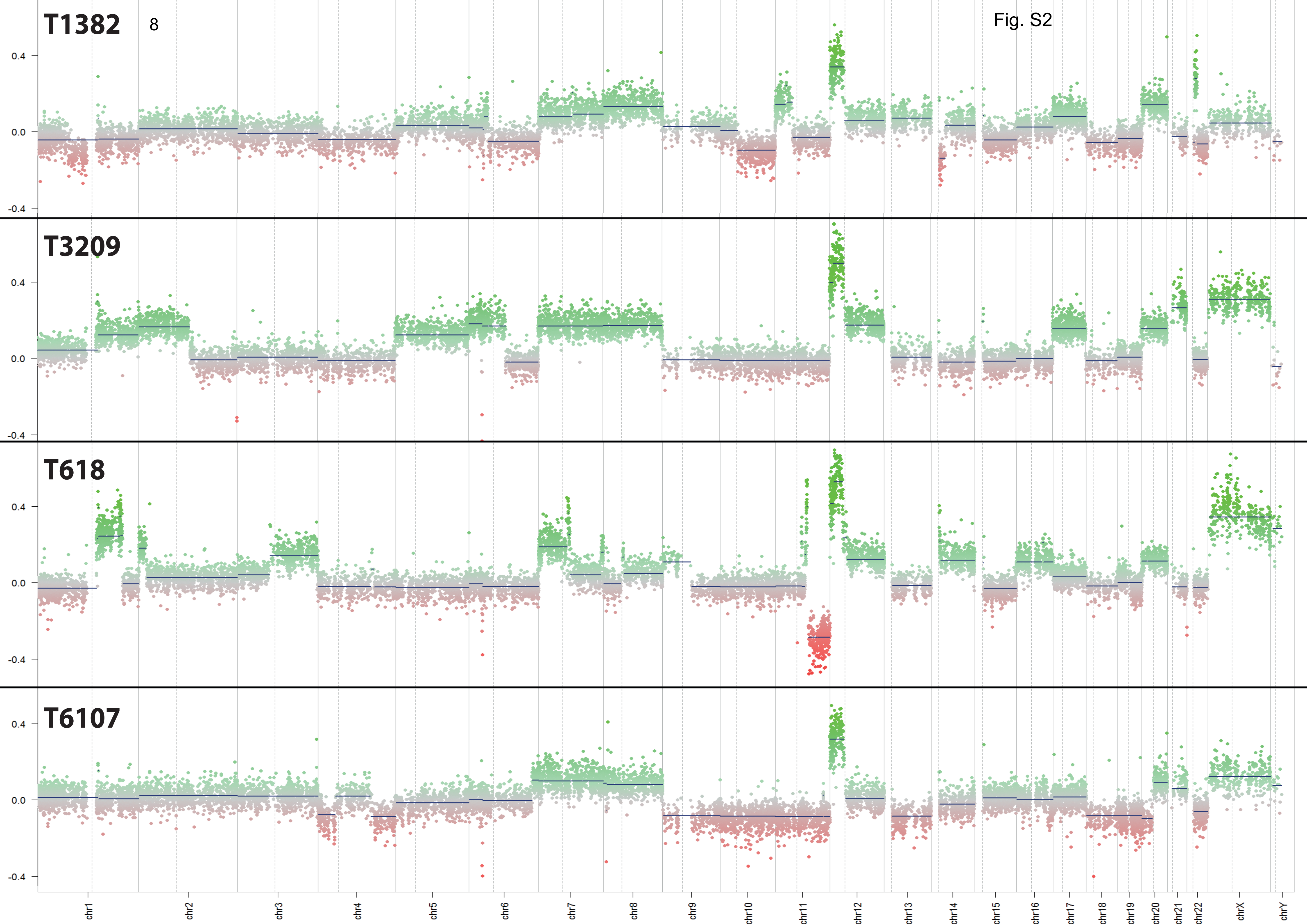
T1382
T3209
T618
T6107
0.4
0.0
-0.4
chr1
chr2
chr3
chr4
chr5
chr6
chr7
chr8
chr9
chr10
chr11
chr12
chr13
chr14
chr15
chr16
chr17
chr18
chr19
chr20
chr21
chr22
chrX
chrY

Fig. S3: Mutational signatures of different series of TGCC. The pooled mutational data of this study (4 cases, 1193 mutations, Supplementary Table S4) and the pooled mutations of the different histologies of TGCC, reported by Taylor-Weiner et al (3), were analyzed for the contribution of the Alexandrov signatures 1-21 using the MutationalPatterns R package (v1.0)(4). The number of cases and mutations are provided, as well as the motif matrix. The mutational spectra for the pooled cases and the contribution of the Alexandrov signatures are shown.

Details:

The trinucleotide profile of single base SNV identified in TGCC was determined and compared to the established set of COSMIC mutational signatures(5-9). The contribution of the BRCA1/2 –related signature 3 is highlighted by the yellow boxes and abundant in the NS and SE cases, only minor for teratomas and absent in the mediastinal tumors. This signature was also absent in spermatocytic tumors (10). Significant contribution to the mutational profile by signatures 16 and 19, with yet undefined etiology, was observed for individual (Figure 1C) and pooled cases The mutational profiles of these germ cell cancers showed hardly any contribution of signatures related to APOBEC mutation and DNA mismatch repair defects, although signature 6 was observed in the pooled NS and SE cases. The age related signatures 1 and 5 contributed only marginally to the profile, likely caused by the young age of patients. No major differences in contributions were observed with the complete set of 30 COSMIC signatures (https://cancer.sanger.ac.uk/cosmic/signatures) or the limited set of 21 signatures.

The significant contribution of the signature 3 is independent of the platform (Illumina / Complete Genomics) or the type of sequencing analysis (exome / WGS) performed, excluding a technical artefact. A recent WGS experiment using the Illumina platform for two new NS cases also revealed a significant contribution of signature 3 (not shown).

Fig. S3

Motif matrix	EMC					Taylor-Weimer et al			
	T1382	T3209	T6107	T618	NS	NS	SE	TE	Media
cases					4	19	18	9	2
Total Mut	300	356	192	345	1193	540	379	585	579
CA A.A	11	5	2	7	25	7	2	2	6
CA A.C	4	8	1	3	16	9	6	12	20
CA A.G	1	2	0	2	5	4	2	1	6
CA A.T	1	3	1	3	8	4	3	5	7
CA C.A	4	7	2	5	18	6	3	6	26
CA C.C	6	3	0	6	15	1	3	15	13
CA C.G	1	1	0	1	3	2	3	5	18
CA C.T	2	4	1	5	12	6	2	15	23
CA G.A	2	1	2	1	6	6	1	7	12
CA G.C	4	2	4	4	14	10	5	12	14
CA G.G	0	1	0	1	2	1	1	1	11
CA G.T	3	2	0	1	6	6	1	4	8
CA T.A	7	4	1	5	17	5	2	1	12
CA T.C	5	2	0	5	12	4	6	5	16
CA T.G	1	1	0	2	4	3	1	4	12
CA T.T	6	1	2	3	12	5	1	5	14
CG A.A	8	6	1	7	22	4	3	8	4
CG A.C	0	2	0	5	7	6	1	1	2
CG A.G	0	9	0	4	13	10	2	3	6
CG A.T	5	8	5	9	27	10	3	4	2
CG C.A	0	4	1	3	8	4	4	1	6
CG C.C	1	1	0	2	4	5	1	1	1
CG C.G	1	0	0	0	1	3	3	2	2
CG C.T	1	3	2	7	13	8	0	5	2
CG G.A	2	0	2	2	6	3	2	0	0
CG G.C	2	2	0	2	6	5	4	1	8
CG G.G	0	1	0	2	3	1	1	1	2
CG G.T	2	2	2	4	10	6	1	3	2
CG T.A	2	3	3	4	12	5	5	5	7
CG T.C	0	1	1	2	4	4	4	3	6
CG T.G	1	3	0	1	5	3	0	2	4
CG T.T	4	9	6	10	29	9	7	7	8
CT A.A	11	19	3	12	45	12	9	4	9
CT A.C	4	3	3	8	18	5	6	4	7
CT A.G	9	11	1	9	30	22	11	25	9
CT A.T	6	6	9	5	26	6	6	6	3
CT C.A	7	6	5	2	20	11	12	14	8
CT C.C	5	4	2	5	16	20	10	40	27
CT C.G	6	8	1	9	24	26	15	31	12
CT C.T	10	10	12	11	43	12	17	35	25
CT G.A	7	8	6	12	33	13	10	12	8
CT G.C	3	6	4	2	15	13	14	14	11
CT G.G	7	4	0	5	16	22	16	19	6
CT G.T	11	6	4	6	27	11	11	15	4
CT T.A	10	10	7	6	33	16	3	12	9
CT T.C	6	5	3	3	17	16	3	26	11
CT T.G	4	10	1	7	22	20	9	28	5
CT T.T	8	11	7	13	39	7	9	12	6
TA A.A	1	4	1	3	9	5	2	1	0
TA A.C	2	1	2	0	5	0	0	3	2
TA A.G	2	2	1	1	6	4	1	1	1
TA A.T	0	5	0	2	7	1	3	0	3
TA C.A	0	0	0	1	1	0	1	3	10
TA C.C	1	4	1	4	10	4	1	8	9
TA C.G	0	4	2	3	9	3	3	6	10
TA C.T	1	5	0	6	12	2	4	7	13
TA G.A	1	1	0	1	3	2	3	5	4
TA G.C	0	2	1	0	3	3	0	1	5
TA G.G	0	3	1	1	5	2	0	4	2
TA G.T	0	1	1	2	4	0	1	1	0
TA T.A	1	7	0	3	11	0	2	2	4
TA T.C	3	4	1	4	12	1	3	0	0
TA T.G	1	1	0	0	2	1	0	1	5
TA T.T	4	2	2	3	11	3	3	0	5
TC A.A	8	6	5	8	27	3	3	4	3
TC A.C	7	1	2	1	11	2	1	1	1
TC A.G	7	1	4	2	14	4	2	6	3
TC A.T	5	8	6	7	26	4	4	6	3
TC C.A	4	3	2	3	12	2	3	4	2
TC C.C	1	3	3	3	10	8	5	3	4
TC C.G	1	3	3	4	11	8	2	5	7
TC C.T	1	8	5	3	17	5	5	13	6
TC G.A	4	4	2	2	12	7	2	3	3
TC G.C	5	1	1	4	11	1	1	2	1
TC G.G	1	1	2	1	5	5	4	2	5
TC G.T	3	1	5	3	12	5	5	1	1
TC T.A	4	1	3	3	11	4	6	3	0
TC T.C	4	6	2	7	19	5	2	2	3
TC T.G	2	5	3	1	11	3	4	1	1
TC T.T	2	5	8	2	17	7	7	6	2
TG A.A	0	4	3	2	9	1	5	2	2
TG A.C	0	1	1	1	3	1	7	0	1
TG A.G	0	0	3	1	4	3	1	7	1
TG A.T	3	2	1	1	7	4	1	2	1
TG C.A	2	1	0	0	3	2	1	0	2
TG C.C	1	0	0	2	3	1	1	2	4
TG C.G	0	0	0	4	4	2	3	7	4
TG C.T	3	5	0	1	9	4	3	1	1
TG G.A	0	0	0	1	1	1	1	0	0
TG G.C	0	1	0	0	1	0	3	3	1
TG G.G	1	0	2	2	5	1	10	1	1
TG G.T	0	0	0	2	2	3	0	1	1
TG T.A	3	3	3	0	9	1	2	1	0
TG T.C	2	2	0	0	4	9	9	2	0
TG T.G	1	2	1	3	7	0	3	0	1
TG T.T	7	4	2	4	17	6	2	7	1

Contribution Alexandrov Signatures 1-21

cases	4	19	18	9	2
Total Mut	1193	540	379	585	579
	EMC	Taylor	Taylor	Taylor	Taylor
	NS	NS	SE	TE	Medi
Sign 1	10.7	20.2	12.3	23.1	4.0
Sign 2	4.6				
Sign 3	45.2	43.7	34.3	7.5	
Sign 4				18.4	54.9
Sign 5	8.9				
Sign 6		14.2	12.4		
Sign 7		8.0		13.0	
Sign 8					9.4
Sign 9	3.4		4.0		
Sign 11					7.0
Sign 12			4.3		
Sign 13					3.3
Sign 16	12.5				
Sign 17				2.5	
Sign 19	13.9	5.0	27.3	28.7	11.2
Sign 20				5.4	6.0
Sign 21		3.0			
Filter2%	0.8	5.8	5.5	1.4	4.2

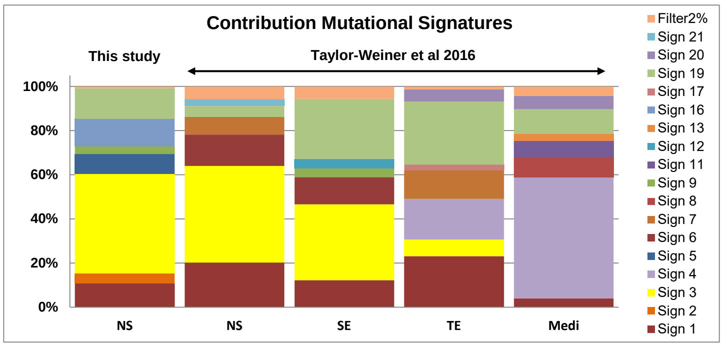

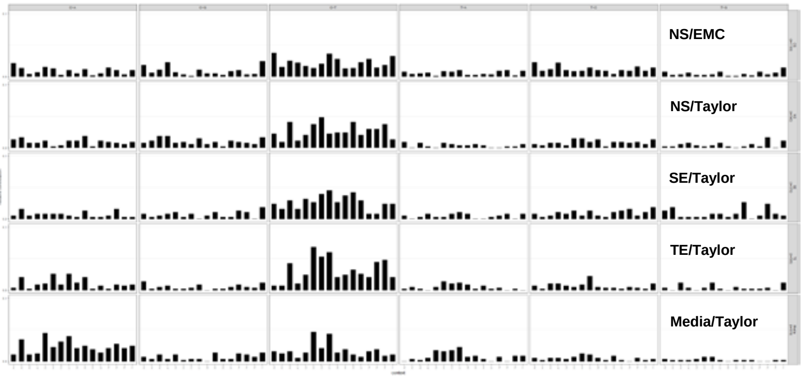

Fig. S4. Methylation status and RNA expression of *BRCA* genes. RNAseq bam files and methylation beta values of the indicated tumor samples were loaded into IGV and images corresponding to the whole gene and of the promoter region of *BRCA1* (panel A) and *BRCA2* (panel B) were exported. CpG methylation beta values above 0.5 are shown as red bars and below 0.5 as green bars.

Details:

CpG residues near the promoters of *BRCA1* and *BRCA2* genes were found to be hypomethylated in all four cases. RNAseq data from casesT6107 and T3209 showed modest numbers of reads derived from both genes. It should be noted that low levels of transcripts from both genes were also observed in normal testis (not shown).

Fig. S4

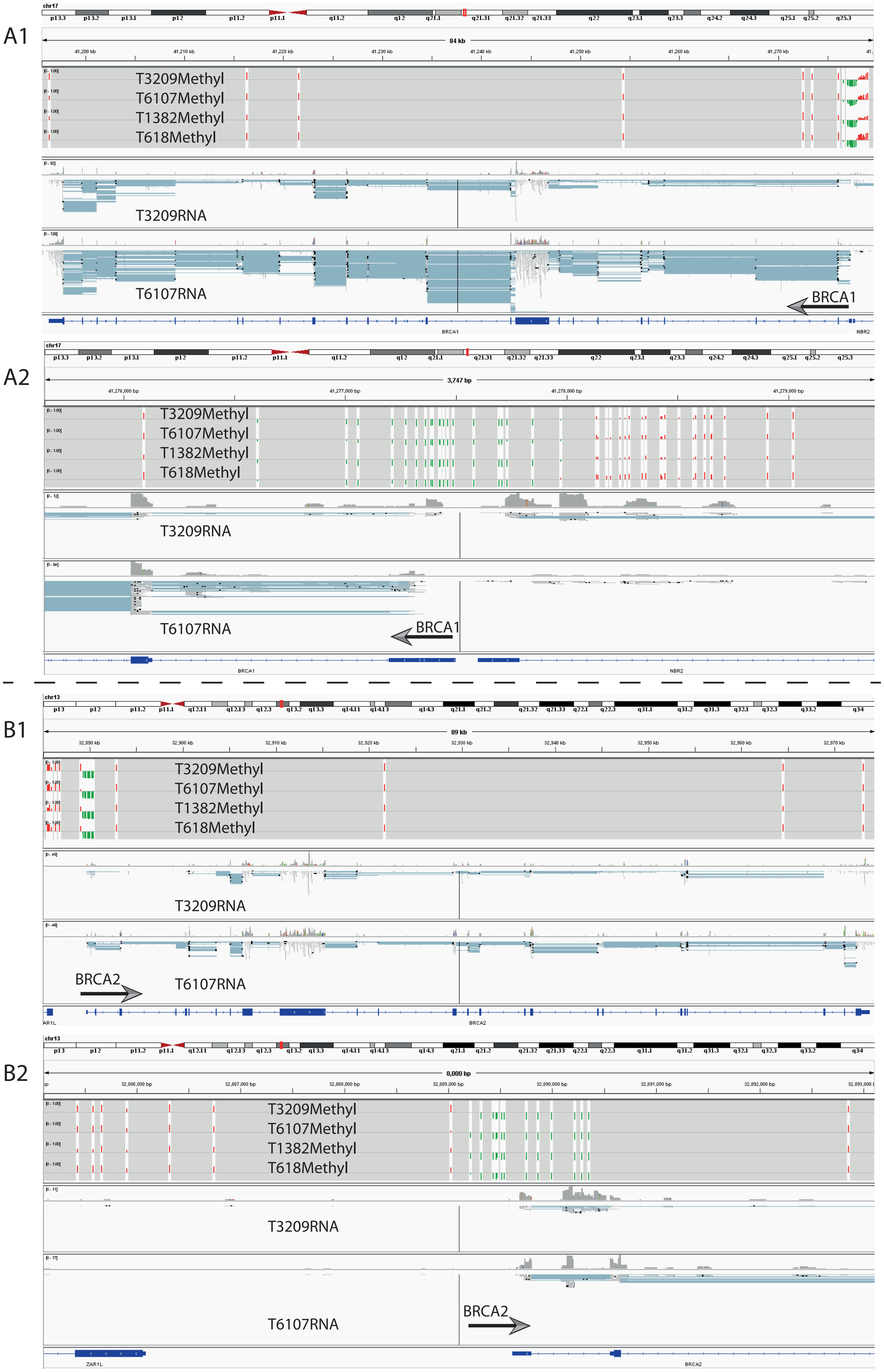
A1
T3209Methyl
T6107Methyl
T1382Methyl
T618Methyl
T3209RNA
T6107RNA
BRCA1
A2
T3209Methyl
T6107Methyl
T1382Methyl
T618Methyl
T3209RNA
T6107RNA
BRCA1
B1
T3209Methyl
T6107Methyl
T1382Methyl
T618Methyl
T3209RNA
BRCA2
T6107RNA
B2
T3209Methyl
T6107Methyl
T1382Methyl
T618Methyl
T3209RNA
T6107RNA
BRCA2

Fig. S5. Examples of images taken during preparation of micro-dissected samples on the PALM. Frozen tissue sections were mounted on slides covered with Polyethylene Naphthalate membrane, stained for alkaline phosphatase (dAP) and counter stained with hematoxylin(11). Capture of specific cells was done after laser excision according to the protocol of the PALM manufacturer. In some cases, adjacent normal cells were removed (see examples: middle panels for GCNIS of case T618) prior to excision of the desired cells. The pictures show examples of the situation before, after laser excision or after transfer to the collection tube.

Details:

Selective staining of GCNIS, EC and EB by dAP allowed for convenient selection of specific cells for laser assisted capture. Teratoma tissue required marking by an experienced pathologist because it was not stained with dAP.

The GCNIS precursor lesions were recovered for three out of the four cases by microdissection after dAP-staining of frozen sections of “normal” adjacent parenchyma (NAP) of the primary tumor. Based on their distribution in the tubule, isolated GCNIS, basal GCNIS (abundant at their niche on the basal membrane) and floating GCNIS preparations were prepared whenever possible(12).

Fig. S5

Representative examples of micro-dissected specimens.

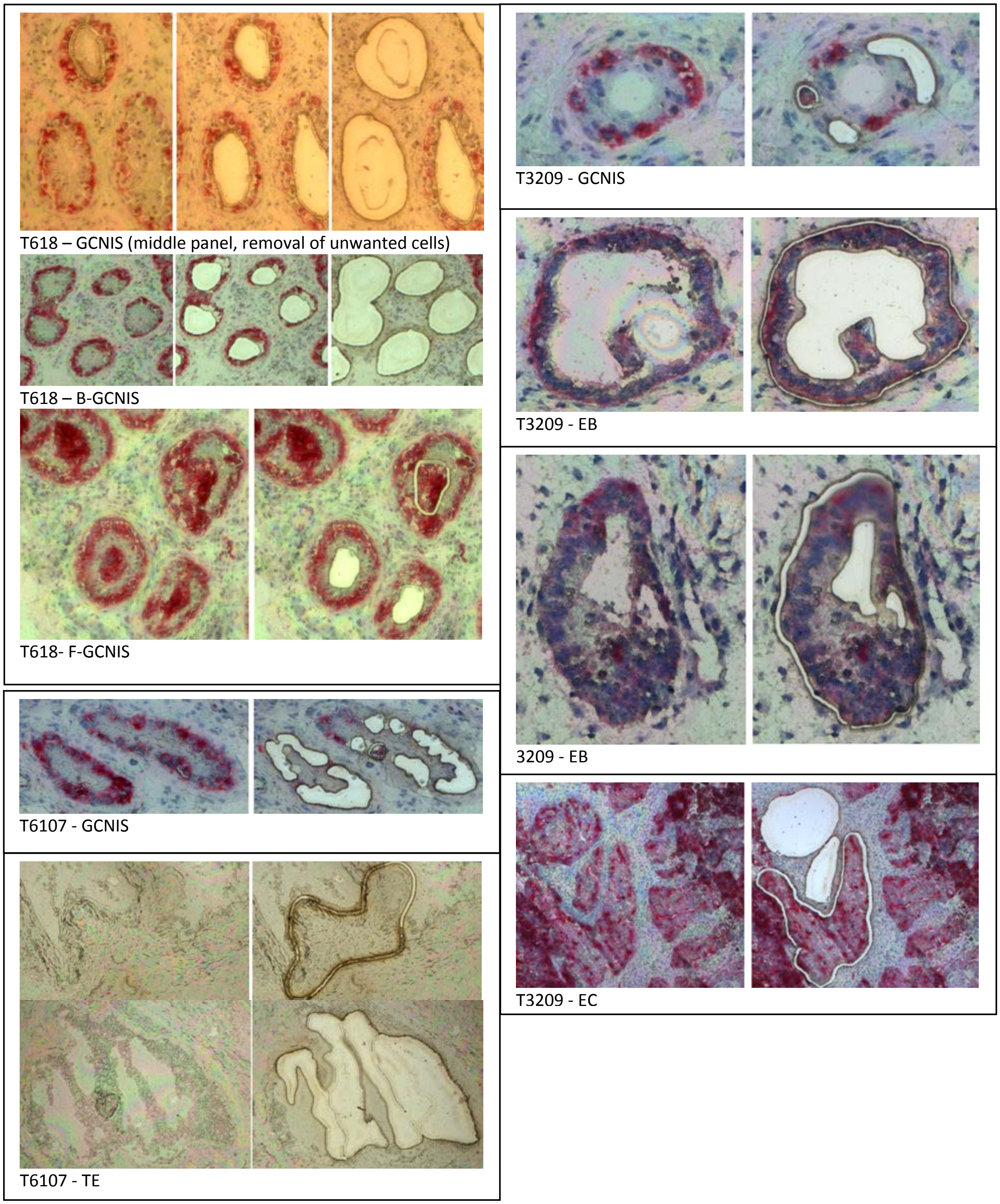

Fig. S6A: T3209

Fig. S6B: T6107

Fig. S6C: T618

Fig. S6D: T1382

Fig. S6. Concordance between WGS and targeted sequencing data. Plotting of targeted sequencing lesser allele frequencies (LAF) of heterozygote SNP positions (red dots) and the read frequencies of SNV (diamonds) in the primary tumor. The bestLAF determined for the targeted regions by WGS for the same DNA sample is indicated (black dots). The position of the targeted amplicons on the genome is indicated on proportional scaled chromosomes (hg19).

Details:

Essentially overlapping LAF profiles were observed for the targeted sequencing and the corresponding segments of the WGS of the same primary tumor DNA samples. The read frequencies for the SNV by targeted sequencing were generally below 40% with notable exceptions for case T618 (chr1 within the amplicon region and chrX) and case T1382 (chr12).

Fig. S6

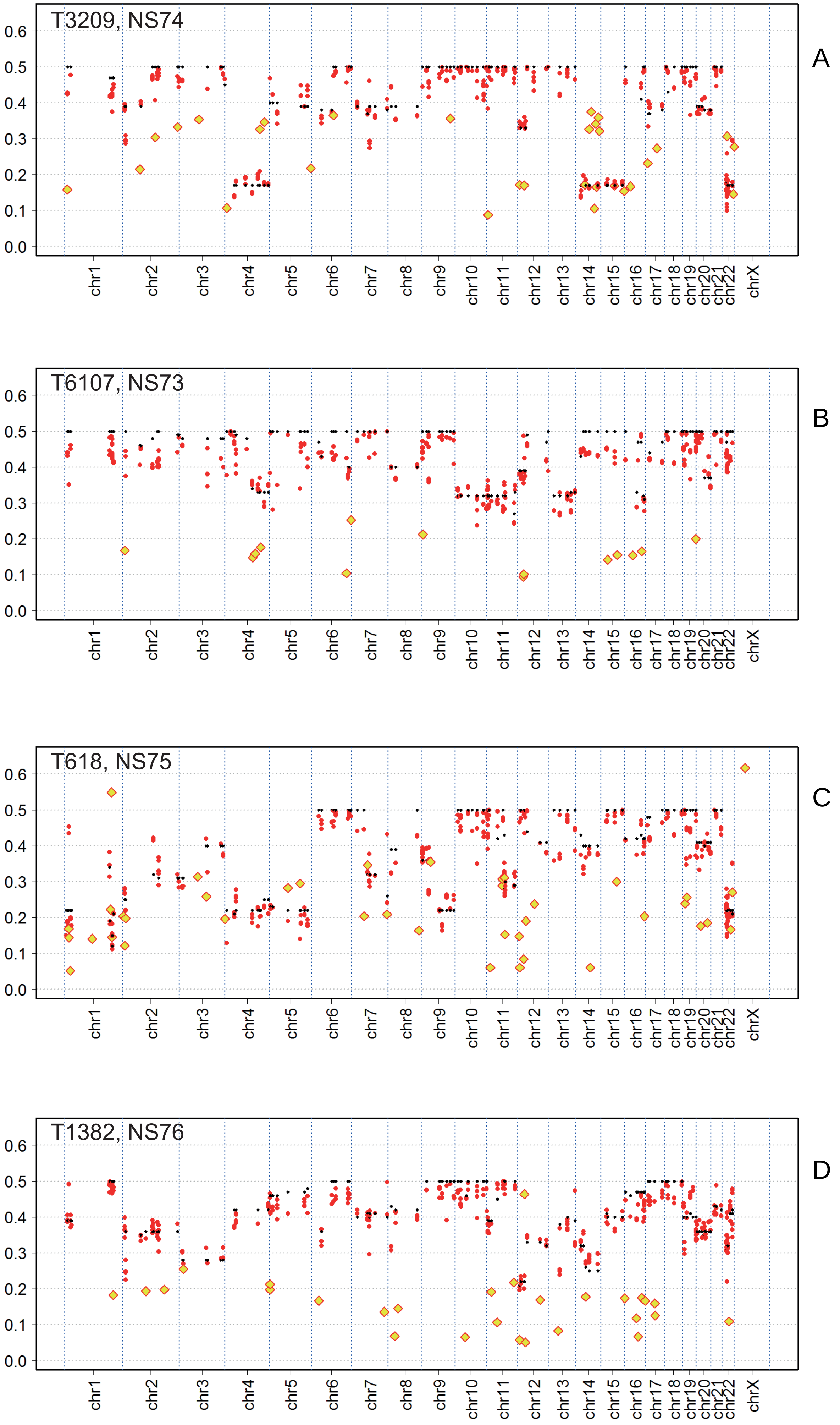
T3209, NS74
A
T6107, NS73
B
T618, NS75
C
T1382, NS76
D
0.0
0.1
0.2
0.3
0.4
0.5
0.6
chr1 chr2 chr3 chr4 chr5 chr6 chr7 chr8 chr9 chr10 chr11 chr12 chr13 chr14 chr15 chr16 chr17 chr18 chr19 chr20 chr21 chr22 chrX

Fig. S7A: T6107

Fig. S7B: T3209

Fig. S7C: T618

Fig. S7D: T1382

Fig. S7. False color plots of amplicon LAF of heterozygote SNPs (top) and somatic alterations (bottom) of samples of individual patients (see also Fig. 2). **Top panel**: SNP LAF data were averaged per amplicon. In addition, WGS Complete Genomic (CG) LAF and relative read coverage (Rel Cov) data of the primary tumor sample for the chromosomal position of the amplicon are shown. SNPS are ordered according to their chromosomal position and chromosome numbers are indicated on the right. Blue indicates balanced alleles, red indicates LOH (color key LAF). **Bottom panel**: SNV read frequencies are presented as fraction of the total read counts and ordered according chromosomal position. Blue indicates absence of SNV and an increase in SNV read frequency is indicated by increased red color (color key SNV). SNV causing amino acid changes are indicated with their gene symbol (see Table 1 and Supplementary Table 6). For T618, only those SNV causing amino acid changes and found in GCNIS have been marked. Structural variants are indicated by an arrowhead and classified as absent (light grey, present in low numbers (grey) or frequent (black). Missing data are in white. On the bottom, a number indicates the time (in months) of collection of that specific specimen after surgical removal of the primary tumor. Sample types have been marked by colored dashed lines (GCNIS: orange, histological components: red and metastases: purple). An (*) indicates that the DNA sample was prepared from paraffin-embedded and formalin-fixed (FFPE) specimen. The red arrow marks a high read frequency SNV on chr12p for case T1382 and for case T6107 on chr19. Color keys for the different categories are shown. Sample information is provided in Supplementary Table S1. Data are available in Supplementary Table S8.

Details:

Primary tumor components: Novel LOH regions involving chromosome arms 9q and 22q were identified in a preparation of EC cells in T6107 (EC21). Similarly, additional LOH was observed on chromosomes 1 and 5 in a single EB of T3209 (Fig. S7B, EB23). The strong over-representation of the short arm of chromosome 12 in the primary NS (Figs. S1, S2 and S7) may have existed with balanced alleles in some cases (T618, T6107). The LAF patterns of some purified histological components showed alterations in

the 12p allelic balance (T6107, EC80 & T3209, EB24), suggesting that the 12p composition may be dynamic during tumor development and progression. The red arrow marked SNV for T6107 (chr19:56131557), resides in a 2kb region between two Zn-finger genes (ZNF865 and ZNF784) and just downstream of a lincRNA. Mutated transcript reads were not observed for this case, nor was aberrant expression of these Zn-finger genes (not shown).

Metastatic samples: The majority of the metastasis samples were derived from FFPE specimens stored for 10-20 years, which did hamper the analyses. Despite drop out of some amplicons and more noisy LAF measurements, data of metastasis samples of two cases shortly (4 months) to long-time (>16 years) after initial surgery and chemotherapy were obtained. For case T1382, a set of SNV, translocations and regions of imbalance appeared preserved in some of the metastases (regions within chromosome 12q, 14, and 22), while novel alterations were also observed (chromosome 6, 9, 11 & the long arm of chromosome 19). Two TE samples (TE24, TE34) and the YST17 sample lack many specific marks present in the primary tumor and its histological components. The molecular profile of the lung metastasis arisen 16 years after initial therapy of case T6107 was distinct from the primary lesion. This YST carried only two of the SNV and a pattern of allelic imbalances with little overlap with the primary NS. These examples show that metastases may share many markers with the primary tumor, in agreement with literature based on microsatellite markers (13, 14), but may also be derived from precursors not identified in the primary tumor.

Precursor lesions: The allele frequency profiles of GCNIS of T6107 and T3209 were mostly balanced and lacked nearly all SNV present in the primary tumor. Low read frequencies (<5%) of 6 SNV for GCNIS of case T3209 may indicate the presence of a minor subpopulation of GCNIS that already acquired some somatic mutations. The GCNIS of T6107 carried a single SNV in high read copies (red arrow). GCNIS representing the different stages of progression (12) were isolated from T618 and carried some of the SNV (>10% of the reads, n=6 out of 40 SNV) and some allelic imbalances (chromosome 4 and 5) also present in the primary tumor. The floating GCNIS (FCIS27) showed the largest shifts in allelic balances. Specific chromosomal translocations and rearrangements were not identified in any of these precursor lesions preparations, although a region on the long arm of chromosome 11 amplified in the primary tumor of T618 already showed a strong allelic imbalance in two of the GCNIS isolates.

Fig. S7A

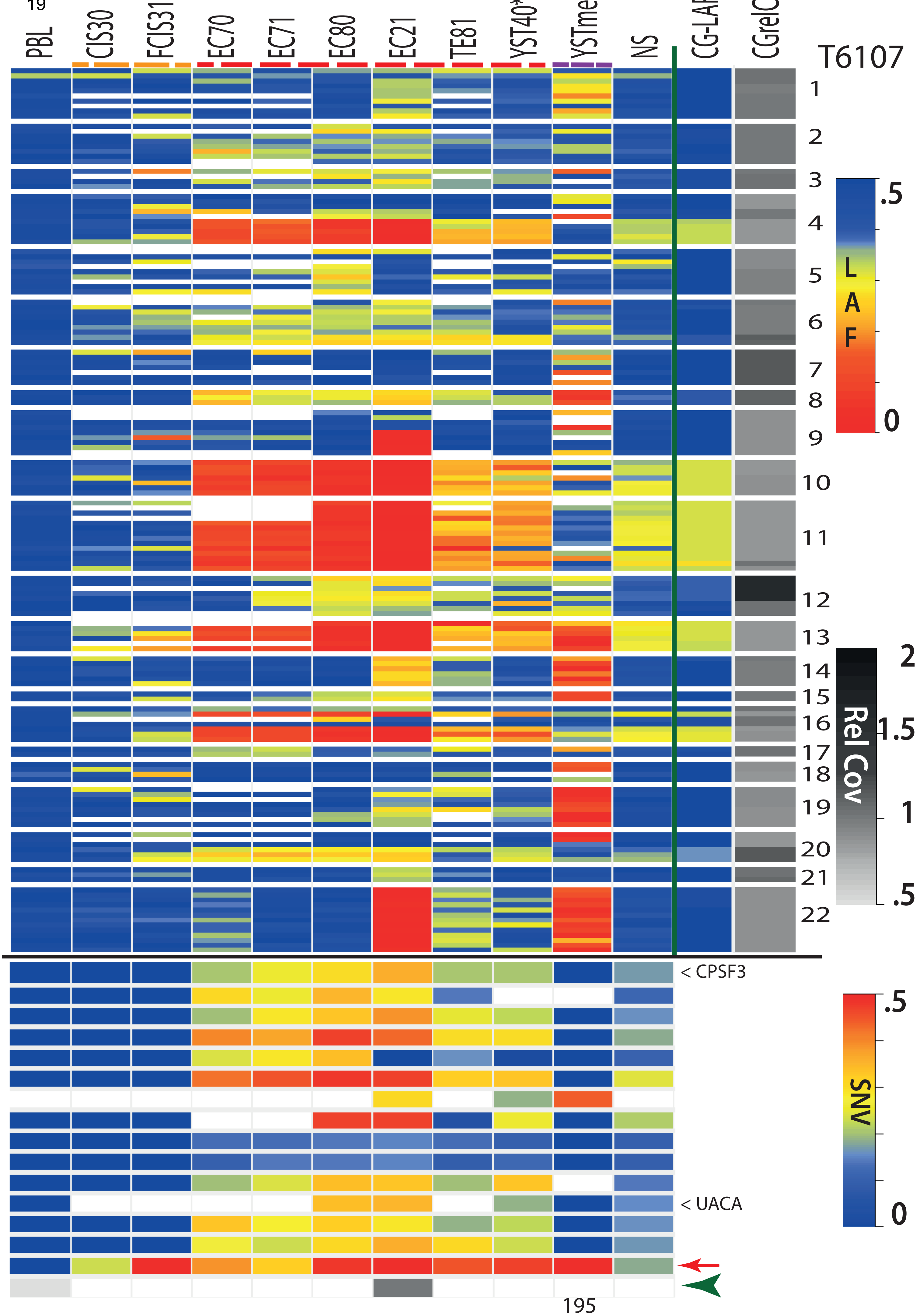
PBL
CIS30
FCIS31
EC70
EC71
EC80
EC21
TE81
YST40*
YSTmeta*
NS
CG-LAF
CGrelCov
T6107
1
2
3
4
5
6
7
8
9
10
11
12
13
14
15
16
17
18
19
20
21
22
.5
L A F
0
2
Rel Cov
1.5
1
.5
< CPSF3
< UACA
.5
SNV
0

Fig. S7B

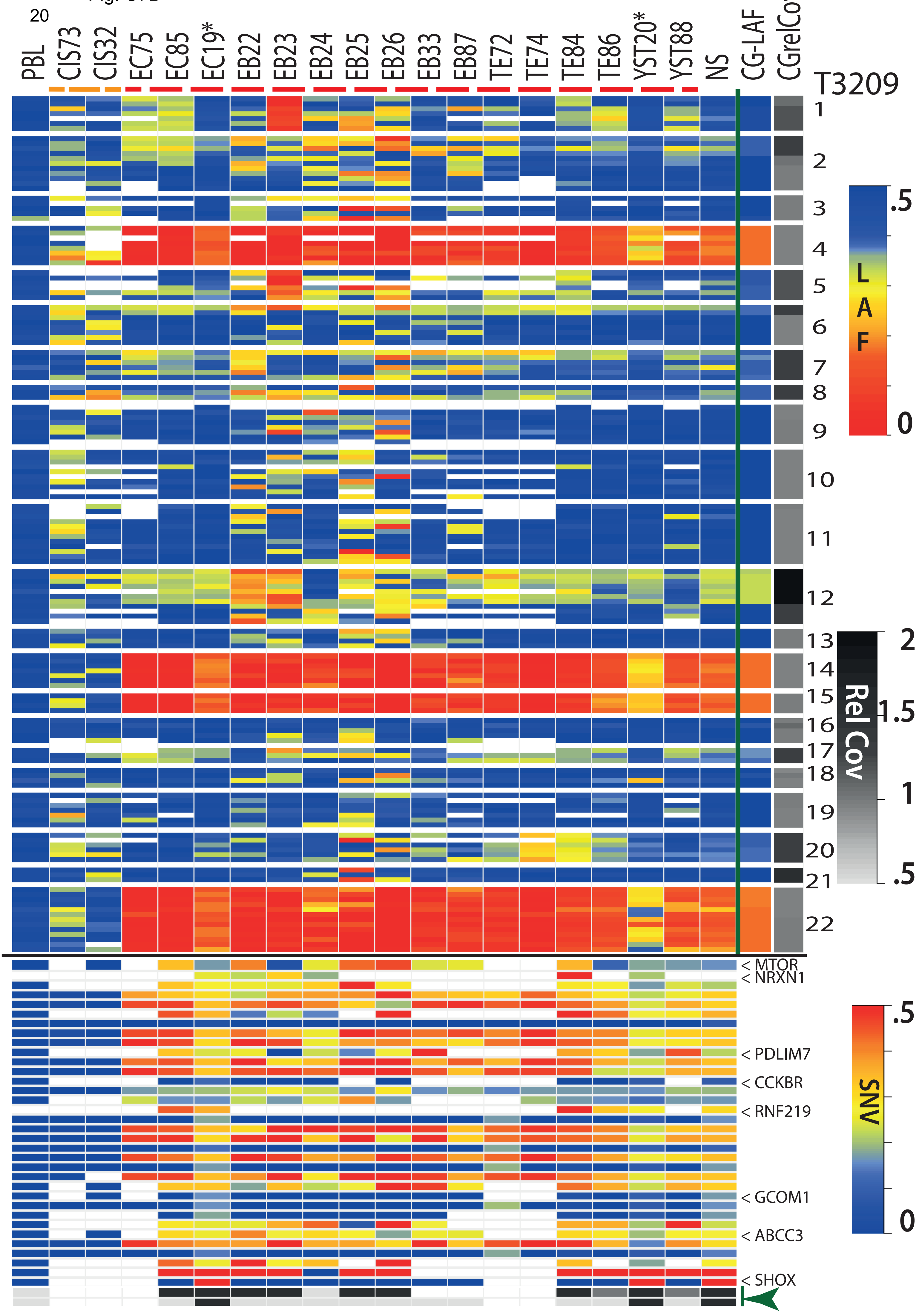
PBL
CIS73
CIS32
EC75
EC85
EC19*
EB22
EB23
EB24
EB25
EB26
EB33
EB87
TE72
TE74
TE84
TE86
YST20*
YST88
NS
CG-LAF
CGrelCov
T3209
1
2
3
4
5
6
7
8
9
10
11
12
13
14
15
16
17
18
19
20
21
22
LAF
.5
0
Rel Cov
2
1.5
1
.5
< MTOR
< NRXN1
< PDLIM7
< CCKBR
< RNF219
< GCOM1
< ABCC3
< SHOX
SNV
.5
0

Fig. S7C

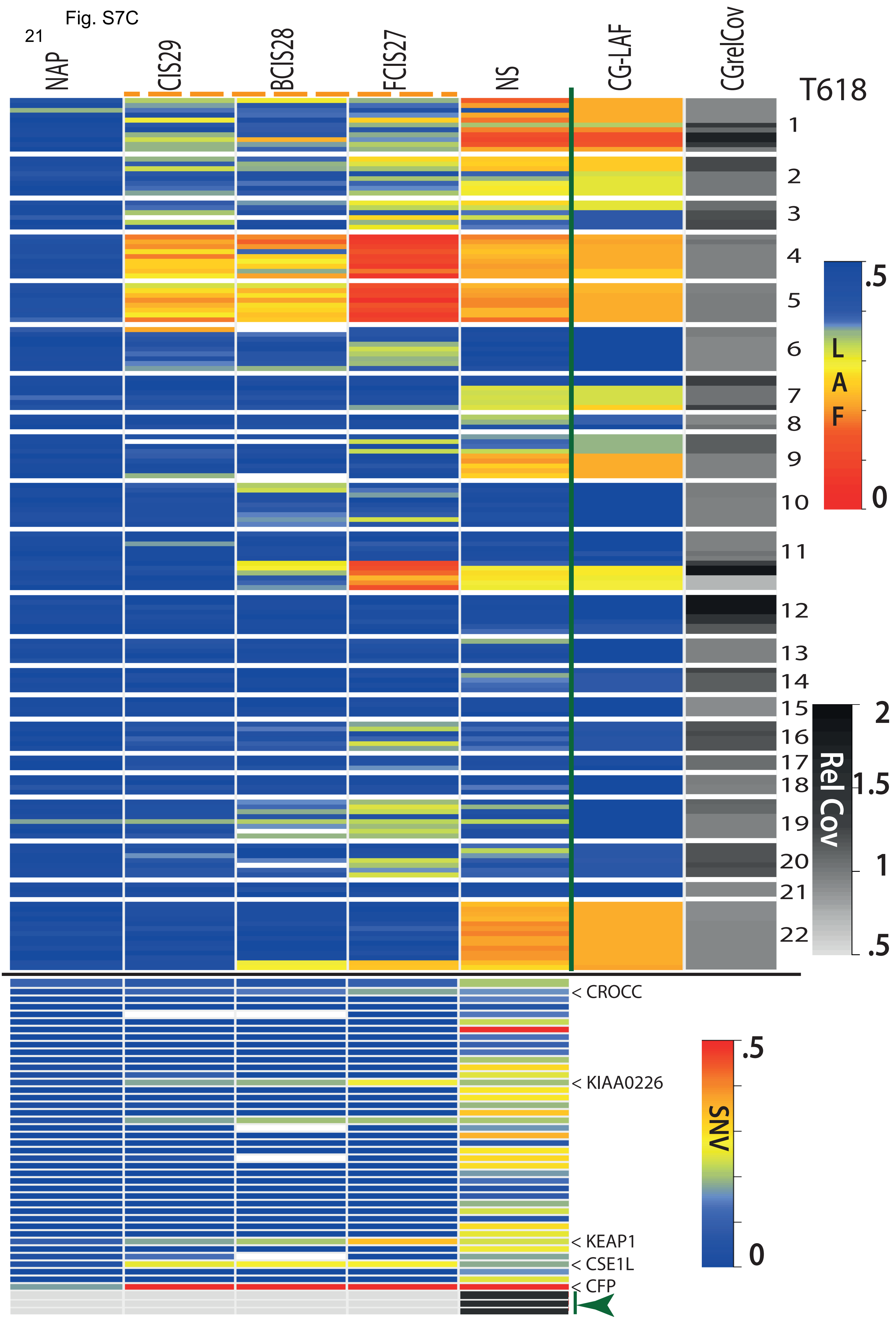
NAP
CIS29
BCIS28
FCIS27
NS
CG-LAF
CGrelCov
T618
1
2
3
4
5
6
7
8
9
10
11
12
13
14
15
16
17
18
19
20
21
22
.5
LAF
0
2
1.5
Rel Cov
1
.5
< CROCC
< KIAA0226
< KEAP1
< CSE1L
< CFP
.5
SNV
0

Fig. S7D

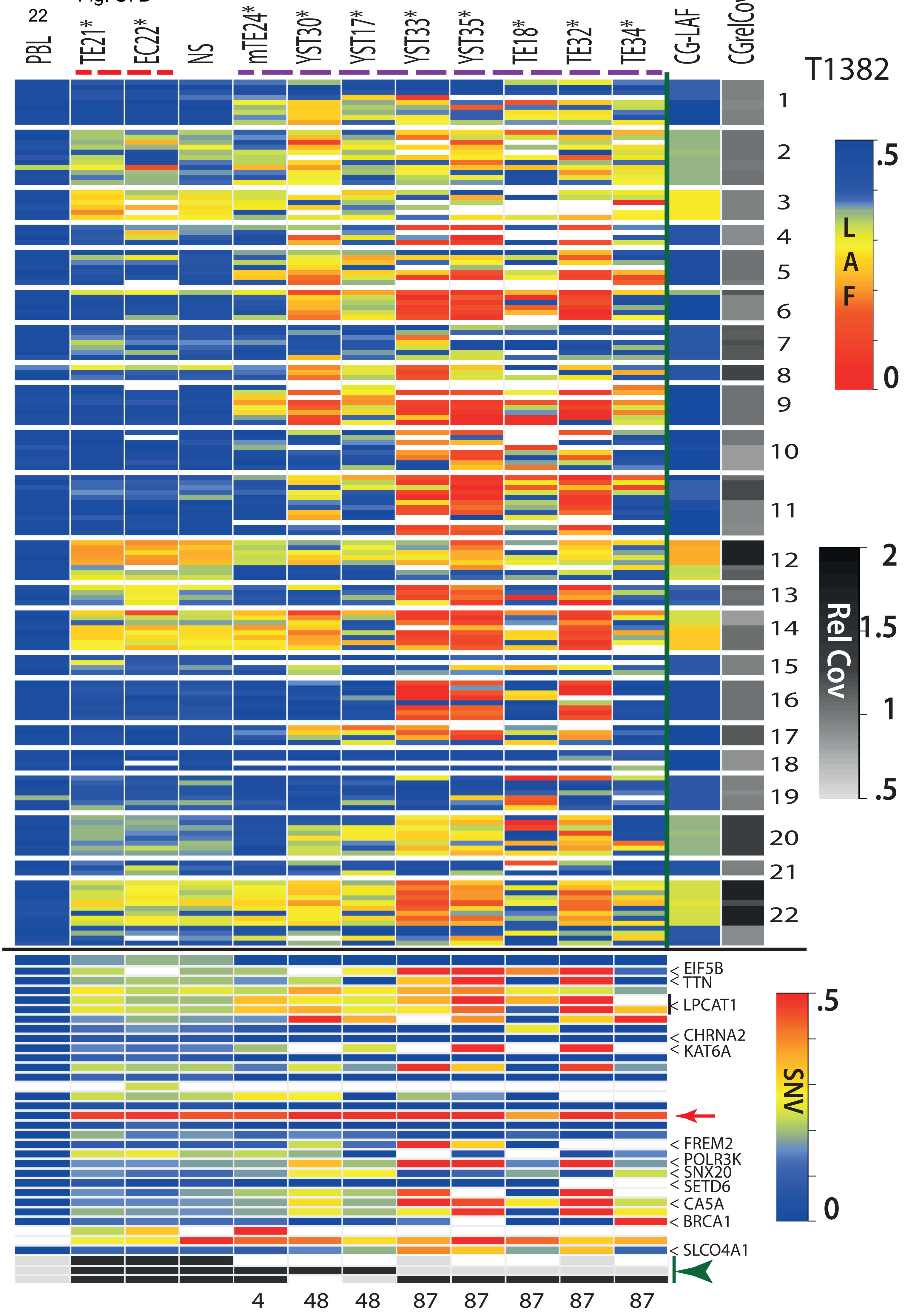
22
PBL
TE21*
EC22*
NS
mTE24*
YST30*
YST17*
YST33*
YST35*
TE18*
TE32*
TE34*
CG-LAF
CGrelCov
T1382
1
2
3
4
5
6
7
8
9
10
11
12
13
14
15
16
17
18
19
20
21
22
.5
L
A
F
0
2
Rel Cov
1.5
1
.5
EIF5B
TTN
LPCAT1
CHRNA2
KAT6A
FREM2
POLR3K
SNX20
SETD6
CA5A
BRCA1
SLCO4A1
.5
SNV
0
4
48
48
87
87
87
87
87

Fig. S8: Loss of different parental alleles in tumor subpopulations. Allelic frequencies of heterozygote positions of selected regions in different samples are presented. In the top panel, different populations of GCNIS are compared with normal and tumor cells (T618) for allelic imbalances on chromosomes 11 and 22. In the boxed regions, different alleles are lost in the GCNIS compared to the primary tumor. The bottom panel shows distinct parental allele losses on chromosome arm 22q in an EC component (EC-21) and the lung metastasis (YSTmeta) of T6107.

Details:

The plots show the allele frequencies of each individual heterozygote SNP, which is the frequency of the variant allele reads of the total of the reference and variant allele reads. A chromosomal region. Remarkably, for case T618 different allelic copies of 11q (immediately downstream of the amplicon region, Fig S1C and S2)), and of the tip of chromosome 22 appear to be lost in the GCNIS (FCIS27/BCIS28) compared to the primary tumor. Similarly, for case T6107, different allelic copies of chromosome arm 22q have been retained by the YSTmeta and the purified EC-21 sample, while the primary tumor appeared balanced for both alleles.

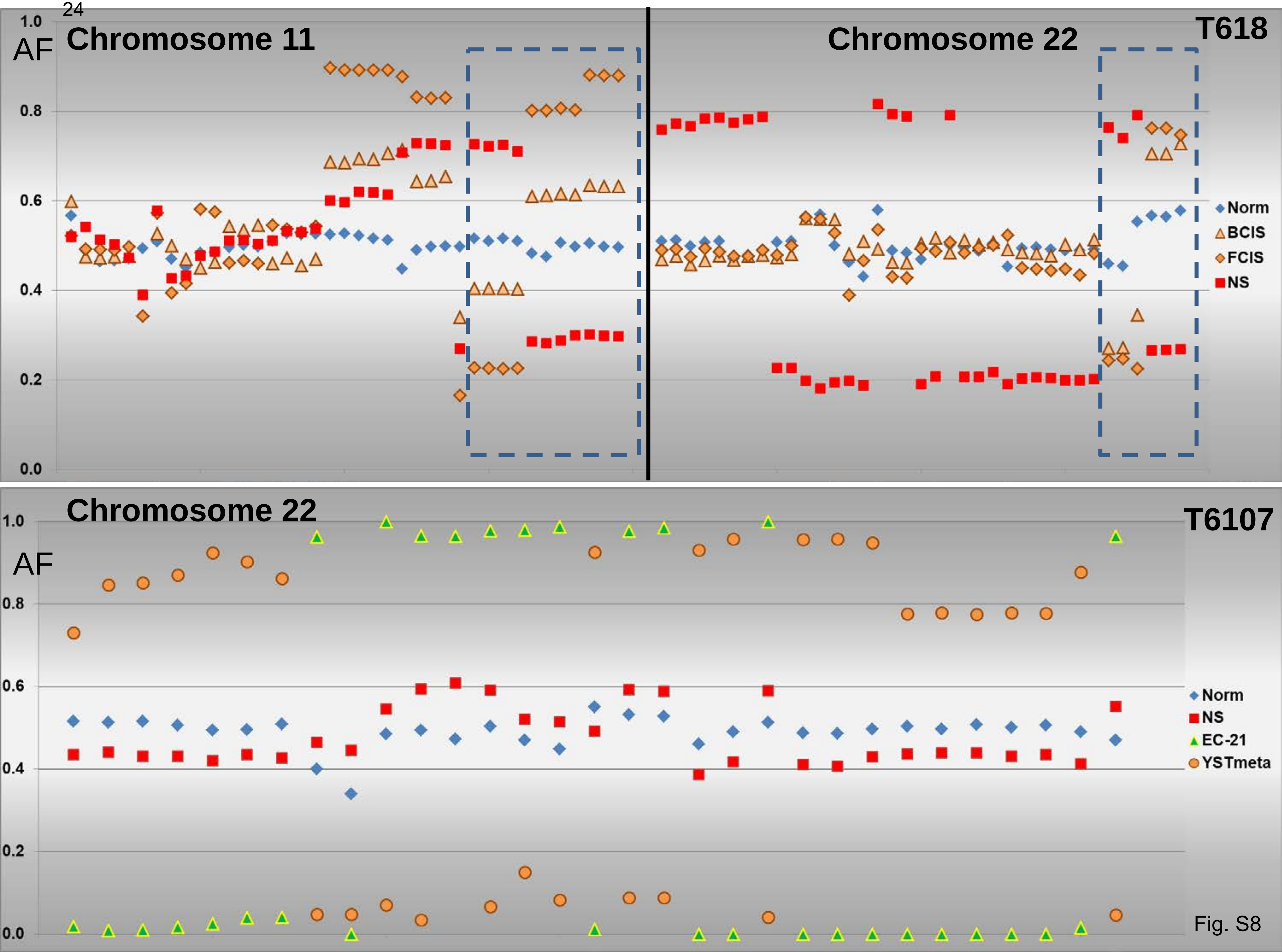
T618
AF
Chromosome 11
Chromosome 22
1.0
0.8
0.6
0.4
0.2
0.0
Norm
BCIS
FCIS
NS
Chromosome 22
T6107
AF
1.0
0.8
0.6
0.4
0.2
0.0
Norm
NS
EC-21
YSTmeta

Fig. S8

Fig. S9: CNA and IHC profiles of metastatic lesions of case T6107. **A**. Immunohistochemistry staining's of FFPE tissue sections of the YST lung metastasis for pan keratin (AE1/AE3/PCK26; VENTANA 760-2595), OCT4 (MRQ10; CELL MARQUE 760-4392), MDM2 (IF2; MILLIPORE MABE340) and TP53 (BP3-11 VENTANA 760-2542) are presented. **B**. Relative copy numbers derived from the methylation profile of metastatic YST DNA of case T6107. Methylation was determined on an Illumina methylationEPIC BeadChip array and processed using the Conumee package analogous to Fig. S2. The inserts show details of a FISH experiment using a red labeled MDM2-specific probe (KBI-10717, Kreatech/Leica Biosystems, Amsterdam). FISH images were visualized using a fluorescent microscope (Leica Microsystems, Rijswijk, The Netherlands). Right panel: a couple of normal diploid cells (arrow head) are detectable while tumor cells show increased copy numbers of the MDM2 region (arrow). The green chromosome 12 centromere probe (D12Z3, KBI-20012G) was not detected properly due to the intense green background signal. After extensive background reduction with an in house plug-in script for FIJI (https://imagej.net/Fiji), regions with normal cells (left panel) and tumor cells (middle panel) are identified. The abundant red MDM2 hybridization signal is clearly detected on the blue stained tumor nuclei (middle panel). **C**. LowPass WGS copy number profile of the retroperitoneal teratoma (15 months after primary surgery) isolated with the DEPArray (41 Keratin-positive and Vimentin-negative cells, see also Fig. S10). The arrow indicates the MDM2 amplified region and the insert shows the strong MDM2 immunostaining of the epithelial tumor cells.

Details:

The methylation-derived chromosome copy number profile of the lung metastasis displayed many novel alterations including amplification of the MDM2 region compared to the primary tumor (Figs. S1&S2). IHC and FISH analysis confirmed the amplification of MDM2. An amplified region including MDM2 was also observed after LowPass WGS of a DEPArray purified cells (15) of a prior lymph node metastasis with the histology of mature TE. The genomic copy number profiles for both metastases displayed many differences among each other as well with the primary tumor (Figs. S1B&S2). Both showed the over-representation of the short arm of chromosome 12.

TP53 mutation was not found using targeted sequencing of the lung YSTmeta genomic DNA (not shown).

Fig. S9

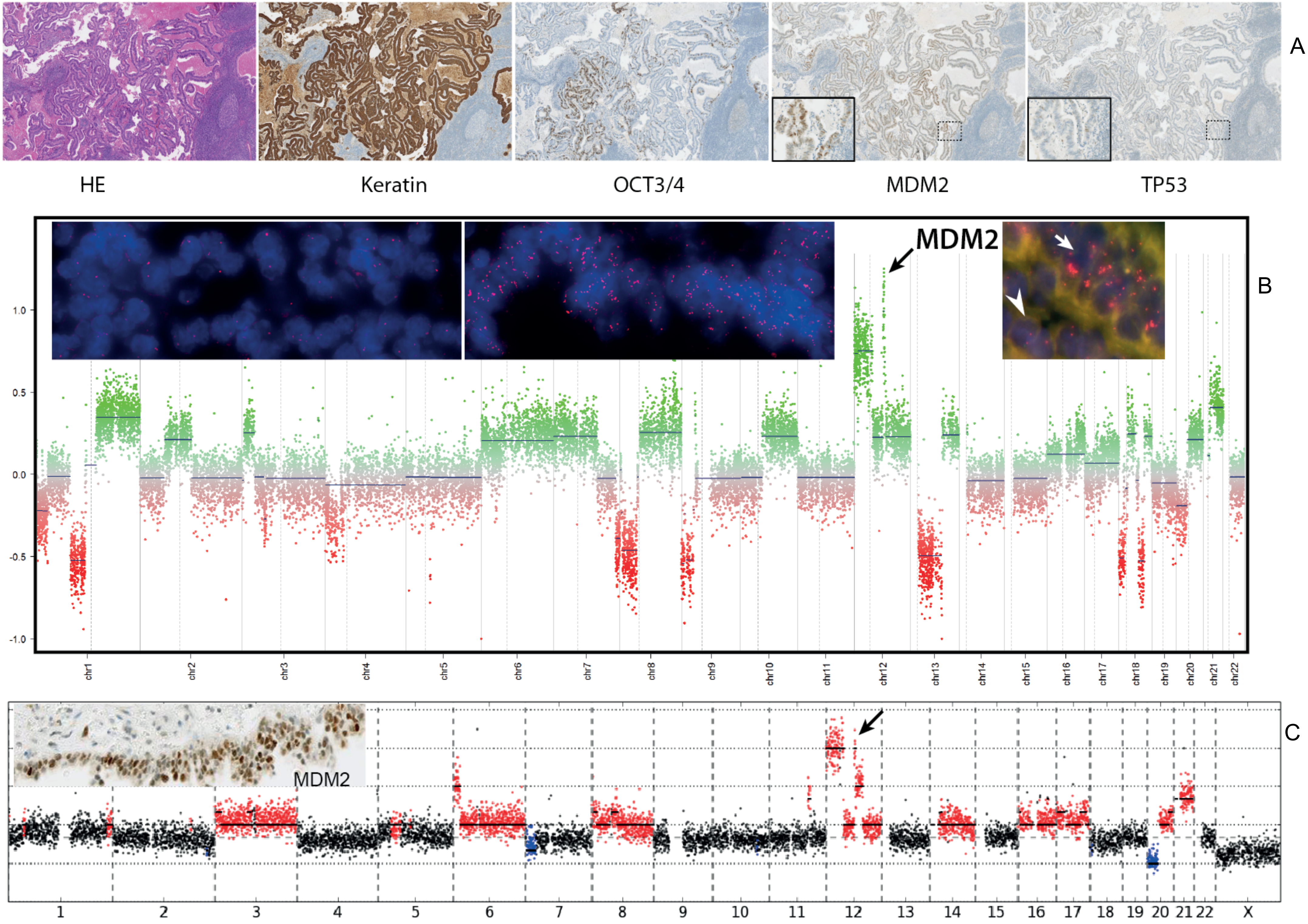
A
HE
Keratin
OCT3/4
MDM2
TP53
B
MDM2
1.0
0.5
0.0
-0.5
-1.0
chr1
chr2
chr3
chr4
chr5
chr6
chr7
chr8
chr9
chr10
chr11
chr12
chr13
chr14
chr15
chr16
chr17
chr18
chr19
chr20
chr21
chr22
C
MDM2
1
2
3
4
5
6
7
8
9
10
11
12
13
14
15
16
17
18
19
20
21
22
X

Fig. S10: Copy number profile derived from loss pass WGS of DEPArray^{TM} purified T618 cells. YST cells of case T618 were purified from two regions of FFPE tissue sections using Keratin (K) -positive and Vimentin (V) -negative staining. GCNIS cells (Vimentin / Keratin double negative and with increased DAPI staining (peak DNA index of 1.44) and Vimentin-positive and Keratin-negative stromal cells were isolated from adjacent normal parenchyma. DNA isolated from these purified cells was subjected to LowPass WGS and relative copy number profiles were generated using Control-FREEC software.

Details:

Additional frozen histological samples of T618 could not be obtained from case T618, but two isolates of YST cells purified using the DEPArrayTM system (15) from FFPE sections showed chromosomal copy number alterations comparable to the primary tumor (Figs. S1&S2).

LowPass WGS of a purified vimentin/keratin double-negative, aneuploidy purified GCNIS sample did not reveal structural alterations, over-representation of chromosome arm 12p or amplification on chromosome arm 11q. Some alterations in the relative copy numbers of chromosomes were already present in these purified cells.

Fig. S10

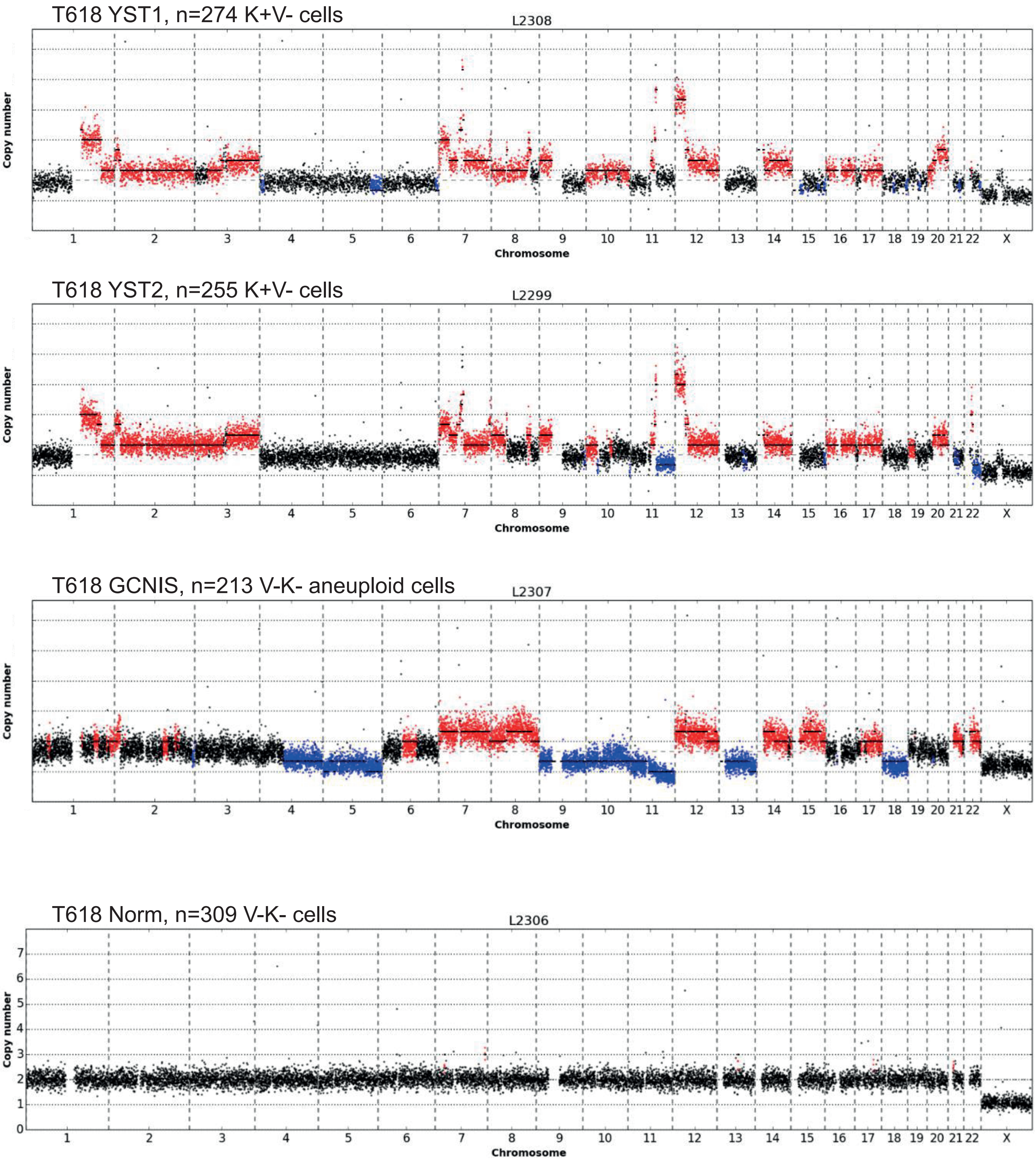
T618 YST1, n=274 K+V- cells
L2308
T618 YST2, n=255 K+V- cells
L2299
T618 GCNIS, n=213 V-K- aneuploid cells
L2307
T618 Norm, n=309 V-K- cells
L2306
Copy number
Chromosome
1 2 3 4 5 6 7 8 9 10 11 12 13 14 15 16 17 18 19 20 21 22 X
0 1 2 3 4 5 6 7

References

1. Hiltemann S, McClellan EA, van Nijnatten J, Horsman S, Palli I, Teles Alves I, et al. iFUSE: integrated fusion gene explorer. Bioinformatics. 2013;29(13):1700-1. Epub 2013/05/11.
2. Hovestadt V, Zapatka M. conumee: Enhanced copy-number variation analysis using Illumina DNA methylation arrays. R package version 1.6.0. http://bioconductororg/packages/conumee/. 2015.
3. Taylor-Weiner A, Zack T, O'Donnell E, Guerriero JL, Bernard B, Reddy A, et al. Genomic evolution and chemoresistance in germ-cell tumours. Nature. 2016;540(7631):114-8. Epub 2016/12/03.
4. Blokzijl F, Janssen R, Van Boxtel R, Cuppen E. MutationalPatterns: an integrative R package for studying patterns in base substitution catalogues. http://biorxivorg/content/early/2016/10/17/071761. 2016.
5. Alexandrov LB, Nik-Zainal S, Wedge DC, Campbell PJ, Stratton MR. Deciphering signatures of mutational processes operative in human cancer. Cell reports. 2013;3(1):246-59. Epub 2013/01/16.
6. Alexandrov LB, Nik-Zainal S, Wedge DC, Aparicio SA, Behjati S, Biankin AV, et al. Signatures of mutational processes in human cancer. Nature. 2013;500(7463):415-21. Epub 2013/08/16.
7. Alexandrov LB, Stratton MR. Mutational signatures: the patterns of somatic mutations hidden in cancer genomes. Current opinion in genetics & development. 2014;24:52-60. Epub 2014/03/25.
8. Chan K, Gordenin DA. Clusters of Multiple Mutations: Incidence and Molecular Mechanisms. Annual review of genetics. 2015;49:243-67. Epub 2015/12/04.
9. Petljak M, Alexandrov LB. Understanding mutagenesis through delineation of mutational signatures in human cancer. Carcinogenesis. 2016;37(6):531-40. Epub 2016/05/22.
10. Giannoulatou E, Maher GJ, Ding Z, Gillis AJM, Dorssers LCJ, Hoischen A, et al. Whole-genome sequencing of spermatocytic tumors provides insights into the mutational processes operating in the male germline. PLoS ONE. 2017;12(5):e0178169. Epub 2017/05/26.
11. Stoop H, Kirkels W, Dohle GR, Gillis AJ, den Bakker MA, Biermann K, et al. Diagnosis of testicular carcinoma in situ '(intratubular and microinvasive)' seminoma and embryonal carcinoma using direct enzymatic alkaline phosphatase reactivity on frozen histological sections. Histopathology. 2011;58(3):440-6. Epub 2011/02/18.
12. Rosenberg C, Van Gurp RJ, Geelen E, Oosterhuis JW, Looijenga LH. Overrepresentation of the short arm of chromosome 12 is related to invasive growth of human testicular seminomas and nonseminomas. Oncogene. 2000;19(51):5858-62. Epub 2000/12/29.
13. Jones TD, Wang M, Sung MT, Zhang S, Ulbright TM, Eble JN, et al. Clonal origin of metastatic testicular teratomas. Clin Cancer Res. 2006;12(18):5377-83. Epub 2006/09/20.
14. Kernek KM, Ulbright TM, Zhang S, Billings SD, Cummings OW, Henley JD, et al. Identical allelic losses in mature teratoma and other histologic components of malignant mixed germ cell tumors of the testis. Am J Pathol. 2003;163(6):2477-84. Epub 2003/11/25.
15. Bolognesi C, Forcato C, Buson G, Fontana F, Mangano C, Doffini A, et al. Digital Sorting of Pure Cell Populations Enables Unambiguous Genetic Analysis of Heterogeneous Formalin-Fixed Paraffin-Embedded Tumors by Next Generation Sequencing. Scientific reports. 2016;6:20944. Epub 2016/02/13.
